# Genome Sequences of Two Strains of *Prototheca wickerhamii* Provide Insight Into the Protothecosis Evolution

**DOI:** 10.3389/fcimb.2022.797017

**Published:** 2022-02-02

**Authors:** Jian Guo, Jianbo Jian, Lili Wang, Lijuan Xiong, Huiping Lin, Ziyi Zhou, Eva C. Sonnenschein, Wenjuan Wu

**Affiliations:** ^1^ Department of Laboratory Medicine, Shanghai East Hospital, Tongji University School of Medicine, Shanghai, China; ^2^ Department of Biotechnology and Biomedicine, Technical University of Denmark, Lyngby, Denmark; ^3^ Department of Laboratory Medicine, Guizhou University The Second Affiliated Hospital of Traditional Chinese Medicine, Guizhou, China

**Keywords:** *Prototheca wickerhamii*, protothecosis, algae, whole genome sequencing, pathogenic

## Abstract

The *Prototheca* alga is the only chlorophyte known to be involved in a series of clinically relevant opportunistic infections in humans and animals, namely, protothecosis. Most pathogenic cases in humans are caused by *Prototheca wickerhamii*. In order to investigate the evolution of *Prototheca* and the genetic basis for its pathogenicity, the genomes of two *P. wickerhamii* strains S1 and S931 were sequenced using Nanopore long-read and Illumina short-read technologies. The mitochondrial, plastid, and nuclear genomes were assembled and annotated including a transcriptomic data set. The assembled nuclear genome size was 17.57 Mb with 19 contigs and 17.45 Mb with 26 contigs for strains S1 and S931, respectively. The number of predicted protein-coding genes was approximately 5,700, and more than 96% of the genes could be annotated with a gene function. A total of 2,798 gene families were shared between the five currently available *Prototheca* genomes. According to the phylogenetic analysis, the genus of *Prototheca* was classified in the same clade with *A. protothecoides* and diverged from *Chlorella* ~500 million years ago (Mya). A total of 134 expanded genes were enriched in several pathways, mostly in metabolic pathways, followed by biosynthesis of secondary metabolites and RNA transport. Comparative analysis demonstrated more than 96% consistency between the two herein sequenced strains. At present, due to the lack of sufficient understanding of the *Prototheca* biology and pathogenicity, the diagnosis rate of protothecosis is much lower than the actual infection rate. This study provides an in-depth insight into the genome sequences of two strains of *P. wickerhamii* isolated from the clinic to contribute to the basic understanding of this alga and explore future prevention and treatment strategies.

## Introduction

The genus *Prototheca* belongs to the predominantly photosynthetic family of the green algae Chlorellaceae but has forfeited the photosynthetic ability (Severgnini et al., 2018; Bakula et al., 2020). All *Prototheca* species possessing colorless plastids evolved from photosynthetic algae that had lost genes related to photosynthesis, yet they retained vestigial plastids (Suzuki et al., 2018). *Prototheca* can cause pathogenic disease in humans and animals with *Prototheca wickerhamii* and *Prototheca zopfii* being the two most common pathogenic species (Kwiecinski, 2015). *P. wickerhamii* has been reported to be the more common human–pathogenic species compared to *P. zopfii*, which infects cattle and dogs (Leimann et al., 2004; Todd et al., 2018). Clinically, *P. wickerhamii* causes protothecosis, usually infects skin and subcutaneous tissue, and can affect both immunocompetent and immunocompromised patients (Todd et al., 2018). In cattle, it has been identified in infected mammary glands (Todd et al., 2018). A previous report described the chain of infection with, *P. wickerhamii*, namely the reservoirs, route of transmission and length of incubation, as unknown or variable (Khan et al., 2018). By 2017, at least 211 cases caused by *P. wickerhamii* had been reported (Bakula et al., 2021). In our laboratory, we have collected 59 *Prototheca* isolates from Chinese clinical patients coming from Shanghai, Beijing, Zhejiang, Sichuan, Jiangsu, Shandong, Jiangxi, Fujian, Guangxi, Guangdong, Yunnan, Chongqing, and Henan. Based on the real-time PCR targeting portion D1/D2 of the 28S rRNA gene, *P. wickerhamii* was found to be the most abundant species in 37 patients with systemic or cutaneous protothecosis.

However, due to the lack of knowledge on the organism and molecular pathogen detection (Khan et al., 2018), protothecosis is difficult to diagnose leading to many patients failing to be treated timely and appropriately (Zeng et al., 2019). Despite the pathogenic potential of *Prototheca* spp., limited scientific knowledge of the genus has hindered the progress on how to prevent or treat infections. Until now, only little genomic data of *Prototheca* exist to support building this understanding. Five nuclear genome sequences are currently available in the NCBI database originating from five *Prototheca* species, namely, *Prototheca ciferrii* SAG2063 (Severgnini et al., 2018), *Prototheca cutis* JCM 15793 (Suzuki et al., 2018), *Prototheca stagnora* JCM 9641 (Suzuki et al., 2018), *Prototheca bovis* SAG 2021 (Severgnini et al., 2018), and *P. wickerhamii* ATCC 16529 (Bakula et al., 2021). However, all the public genomes (except for the one of *P. wickerhamii*) are draft genomes assembled from next-generation sequencing data, which limits the detailed genomic protothecosis research. Using the first closed genome, Bakula et al. could pioneer in describing the putative genes associated with protothecosis and disease development in *P. wickerhamii* (Bakula et al., 2021). Many genes associated with fungal pathogenicity, some potential virulence factors, and putative genes involved in protothecosis were identified in the *P. wickerhamii* genome (Bakula et al., 2021).

Besides its role as pathogen, *Prototheca* also represents an important model to study algal evolution due to the loss of photosynthesis. The taxonomic position of *Prototheca* as well as the number of *Prototheca* species have been debated for a long time (Jagielski et al., 2019). The phylogeny of *Prototheca* has been revised significantly several times because of more information becoming available including phenotypic, chemotaxonomic, and molecular data (Jagielski et al., 2019). The genus *Prototheca* along with *Helicosporidium* and *Auxenochlorella* belong to the green algal family of Trebouxiophyceae (Suzuki et al., 2018), with the polyphyly being reinforced with plastid genome-based phylogeny (Bakula et al., 2020). However, a study of the phylogenetic relationships of *Auxenochlorella protothecoides* and 23 *Prototheca* strains demonstrated inconsistencies with the molecular phylogenetic analyses based upon complete 16S and partial 23S plastid rDNA sequences (Ewing et al., 2014).

In order to investigate the evolution of *Prototheca* and the genetic basis for its pathogenicity, we herein sequenced two clinical isolates of *P. wickerhamii*, analyzed their phylogeny within the green algae, and identified genes potentially involved in the pathogenicity.

## Methods

### Sampling and Genomic DNA Extraction

The *P. wickerhamii* strain S1 (mucoid colony) was isolated from a case of an 85-year-old patient who had suffered from cutaneous infections in Shanghai, China. The *P. wickerhamii* strain S931 (rough colony) was isolated from a case of a 24-year-old patient who had suffered from multiple cutaneous infections in Shanghai, China (**Figure S1**). For macroscopic observation of colonies, each strain was streaked out on Sabouraud dextrose agar medium and incubated at 35°C for 3 days. Wet specimens were observed under a differential interference contrast microscope. The strains were harvested from agar culture medium and washed with distilled water, and then the genomic DNA was extracted with the CTAB method as described previously (Jagielski et al., 2017).

### Library Preparation and Sequencing

Extracted genomic DNA (gDNA) was prepared for Illumina sequencing as libraries with insert sizes of about 350 bp using the HiSeq X Reagent Kit v2. An amplification-free approach was performed for 150-bp paired-end reads following the manufacturer’s protocol (Illumina) and sequenced on a NovaSeq 6000 Sequencing platform. The PCR duplicates, adapter, N content (N content >5%), and low-quality (quality value ≤10, low-quality base >20%) reads were excluded using SOAPnuke version 1.5.3 (Chen et al., 2018). Then, the clean reads were retained for genome survey. A total of 10 μg gDNA was used to select fragment sizes (>10 kb) with a BluePippin BLF7510 cassette (Sage Science, Beverly, MA, USA). The standard Oxford Nanopore Technologies library prep protocol was applied with a ligation sequencing kit SQK-LSK109. Then, DNA fragments were end-repaired, recovered, purified, and sequenced on the GridION X5 platform. The raw reads were removed with a Q value of <7 and a minimum read length <5,000 bp. The retained clean reads were applied to assemble the genome.

### Genome Survey and Assembly

To obtain the genome size, heterozygosity, and repeat content, Illumina 150-bp paired-end reads were initially used to estimate the *P. wickerhamii* genome characteristics using a 17 K-mer by JellyFish (Marcais and Kingsford, 2011) and GenomeScope (Vurture et al., 2017). The quality-filtered Nanopore long reads were assembled using CANU v1.8 (Koren et al., 2017) with the parameters corOutCoverage = 40, and minReadLength = 1000 and Necat (Chen et al., 2021) with parameters –ctg_min_length=1000 and –coverage=40, respectively. The primary contigs were polished with raw Nanopore long reads using Racon v1.3.3 (three rounds) (Vaser et al., 2017) and Medaka v0.7.1 (one round) (https://github.com/nanoporetech/medaka). Subsequently, the Illumina short insert size reads were mapped to the polished consensus sequence using BWA-mem (v0.7.17) (Li and Durbin, 2009) and applied to correct the assembled genome by Pilon (v1.23) (Walker et al., 2014) with the default settings.

### Genome Annotation and Functional Annotation

The assembled genome was annotated for repeats and genes. Two kinds of methods including homology-based and *de novo* methods were performed in repeat annotation. Firstly, in the homology-based method, the repeat sequence database of RepBase v21.12 (http://www.girinst.org/repbase) (Bao et al., 2015) was applied to identify the similarly repetitive sequences using RepeatModeler v2.0 (Flynn et al., 2020) (http://www.repeatmasker.org/RepeatModeler/)and LTR_FINDER v1.07 (Xu and Wang, 2007) (http://tlife.fudan.edu.cn/ltr_finder/). The *de novo* method was processed by RepeatMasker 3.3.0 (Saha et al., 2008) to predict the repetitive sequences. Three different algorithms (homology-based prediction, RNA-Seq data-based prediction, and *ab initio* prediction) were combined to predict the gene structure. In *ab initio* prediction, the coding regions of genes were predicted with a repeat-masked sequence using AUGUSTUS v3.2.3 (Stanke et al., 2006) and SNAP (Korf, 2004). A total of 9 published homology protein sequences from *Arabidopsis thaliana*, *Auxenochlorella protothecoides*, *Chlamydomonas reinhardtii*, *Chlorella variabilis*, *Coccomyxa subellipsoidae*, *Helicosporidium* sp., *Micractinium conductrix*, *Trebouxia* sp. A1-2, and *Prototheca wickerhamii* type strain ATCC 16529 were aligned to the assembled genome using *TBLASTn* (Kent, 2002). The transcriptome data of *P. wickerhamii* S1 and S931 (see below) were mapped to the genome sequences through *BLAST* and *PASA* software. Finally, the three kinds of evidence used for gene prediction and annotation were integrated with the MAKER (Holt and Yandell, 2011) pipeline. The mitochondrial (mtDNA) and plastid (ptDNA) genomes were annotated with the software of Genes of Organelle from the Reference sequence Analysis (AGORA) (Jung et al., 2018).

For functional annotation of protein-coding genes, protein sequences were aligned to five databases including NR (NCBI non-redundant protein), SwissProt (http://www.gpmaw.com/html/swiss-prot.html), KEGG (http://www.genome.jp/kegg/), KOG (Koonin et al., 2004), and TrEMBL (http://www.uniprot.org) using BLASTp with an E-value of 1 E−5. The protein motifs and domains were identified with *InterPro* (Mulder and Apweiler, 2007) and retrieved using Gene Ontology (GO) (Ashburner et al., 2000) terms. Benchmarking Universal Single-Copy Orthologs (BUSCO V5) (Simao et al., 2015) with the chlorophyta_odb10 database was used to investigate the quality of the genome assembly and gene annotation. To predict putative pathogenicity-related genes, the Pathogen-Host Interaction (PHI) database (Urban et al., 2020) was used to align with protein sequences using BLASTp with an E-value (1 E−5) cutoff.

### RNA Extraction and RNA-Seq Data Analysis

A total of six samples (two *P. wickerhamii-*type strains, S1 and S931, with three biological replicates each) of RNA were obtained from cultured cells using the TRIzol reagent (Invitrogen, USA) following the manufacturer’s protocol. The algal cells were firstly treated with DNase I to remove DNA contamination, and then oligo(dT) magnetic beads were used to enrich total RNA. The library construction methods were consistent with previous reports (Jian et al., 2017). Finally, the constructed libraries were sequenced with 150-bp paired-end on a NovaSeq 6000 platform. Raw reads were preprocessed using SOAPnuke version 1.5.3 (Chen et al., 2018). The clean reads were then mapped to the reference genome (the newly sequenced genome of S1) to acquire the position information and the unique reads feature of the sequenced samples. The FPKM value of each gene was calculated using Cufflinks (Trapnell et al., 2010). The differentially expressed gene (DEG) analysis were performed with DESeq2 (Love et al., 2014). The false discovery rate (FDR) ≤0.05 and fold change ≥2 were used as the threshold to identify the genes significantly differentially expressed between the replicates.

### Gene Family and Phylogenomic Analysis

The single-copy orthologous genes were identified with comparative analysis to examine the conservation of the two newly sequenced genomes and 11 published algae species using OrthoMCL (v2.0.9) (http://orthomcl.org/orthomcl/) (Li et al., 2003). The gene sets of the 11 genomes were processed in two steps. The genes encoding proteins less than 50 amino acids in length were excluded and the longest transcript was retained when there were several spliced transcripts in a gene. The similarity of protein sequences was evaluated by all-versus-all BLASTp (v2.2.26) with an e-value threshold of 1e-10 (Altschul et al., 1990). Single-copy orthologues were extracted from the gene clustering result, and the alignment was performed by the MUSCLE program (v3.8.31, http://www.drive5.com/muscle/) with default parameters (Katoh and Standley, 2013). The phase 1 sites of all single-copy orthologous genes were extracted and concatenated to one super-gene for the phylogeny tree using RAxML (v 8.2.12). Then, the species of *Micromonas commoda* was rooted as outgroup using TreeBest (https://github.com/Ensembl/treebest). The divergence time of species was calculated with MCMCTree module in PAML (v 4.9, http://abacus.gene.ucl.ac.uk/software/paml.html). Three time-calibrated points were derived from the TimeTree database (http://www.timetree.org/): *M. commode*–*C. reinhardtii* (792.4–1,019.6 Mya), *O. tauri*–*C. reinhardtii* (781.1–803.1 Mya), and *C. reinhardtii*–*C. variabilis* (586.9–604.9 Mya). The expansion and contraction analysis of gene families were performed with CAFE (v3.1) (De Bie et al., 2006).

### Comparative Analysis

To test the consensus and variation among the *P. wickerhamii* genomes, the newly and previously assembled genomes were compared with each other using NUCmer 3.1 (MUMmer 3.23 package) (Delcher et al., 2003) and the completeness comparison was performed by MUMmerplot 3.5 (MUMmer 3.23 package) (Delcher et al., 2003) on the NUCmer results after filtering to 1-on-1 alignments and allowing rearrangements with a 1-kbp-length cut.

### Ethical Approval

This study was approved by the ethics committee of Shanghai East Hospital, Tongji University School of Medicine (No. 2020-163). The need for informed consents was waived by the Clinical Research Ethics Committee.

## Results

### Genomic Characterization of *P. wickerhamii* and Genome Assemblies

To obtain the genome characteristics including genome size, heterozygosity, and repeat content, about 4.54 and 4.55 Gb of clean data with Illumina short insert size was generated for *P. wickerhamii* strains S1 and S931, respectively (**Table S1**). Both of the Q20 values of clean reads were higher than 98%, and the Q30 value was approximately 95%. Using jellyfish and GenomeScope (Kmer = 17), the estimated genome sizes were about 18.65 and 17.97 Mb for strain S1 and S931, respectively (**Figures S2, S3**). The heterozygosity was 2.69% for S1 and 3.96% for S931. The estimated genome size was a little higher than the one of the published genome of *P. wickerhamii*-type strain ATCC 16529 with 16.7 Mb. Using Nanopore sequencing on the GridION, in total, 1,328,625 and 681,088 raw reads were generated for S1 and S931, respectively. After filtering out the low-quality reads (Q value of <7 and minimum read length <5,000 bp), a total of 551,989 and 542,630 reads were retained for subsequent assembly. The total clean data were about 4.55 Gb with an average length of 8,235 bp and a maximum length of 153,066 bp for S1. For S931, with a total of 4.90 Gb, the average length was 9,035 bp and the maximum length was 149,724 bp (**Table S2**). The clean Nanopore long reads were used to obtain two primary genome sequences with Canu and Necat (**Tables S3, S4**), and the Necat-based genomes were used for downstream analyses.

### Nuclear Genome Acquirement and Annotation

**Figure 1:**
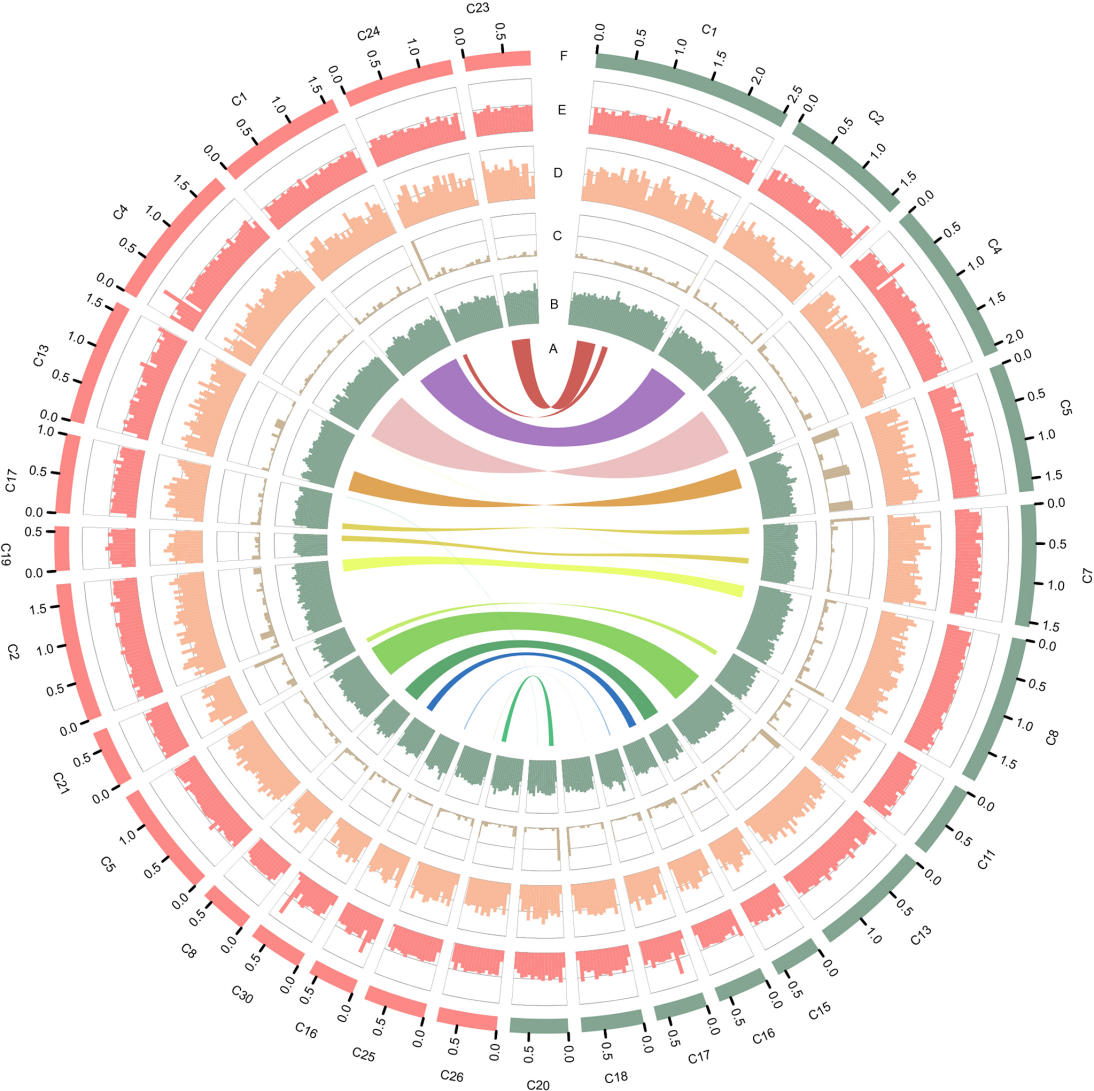
Characteristics of the two types of *P. wickerhamii* genomes. Distribution of genomic features of the *P. wickerhamii* genomes for strain S1 (red) and strain S931 (green). From inside to outside: **(A)** syntenic gene blocks, **(B)** GC content, **(C)** TE density, **(D)** gene number, **(E)** gene length, and **(F)** Contig (>500 kb).

The mitochondrial (mtDNA) and plastid (ptDNA) genomes were obtained from the assembled sequences (**Figure S4** and **Table S5**). After filtering the mtDNA and ptDNA genome sequences out, the length of the final nuclear genome of S1 was approximately 17.57 Mb with 19 contigs (**Table S6**). A final nuclear genome of 17.45 Mb with 26 contigs was generated for S931 (**Table S7**). For strain S1, the contig N50 was 1,639,047 bp with 98.25% of the genome present on 13 contigs, each larger than 500 kb. For S931, the contig N50 was 1,406,360 bp with 93.47% of sequences assembled in 15 contigs of more than 500 kb in size. The characteristics of the two strains of *P. wickerhamii* genomes are shown in **Figure 1**. The assembled genomes suggested that the long contigs were close to the chromosome level. The GC content of both genomes was approximately 64%. The repetitive sequences were initially annotated with homology-based and *de novo* methods. 3.11% and 2.49% of repetitive sequences were identified in S1 and S931, respectively, which might be the reason for *P. wickerhamii* genomes being the smallest and most compact microalgal genomes (**Table S8**). These results are a little higher than that of 2.25% in the genome of *P. wickerhamii* strain ATCC 16529. A total of 5,694 and 5,704 protein-coding genes were predicted in *P. wickerhamii* strains S1 and S931, respectively (**Table 1**), and thereby were slightly less than in strain ATCC 16529. The high quality of the genome and gene annotation was confirmed with the BUSCO V5 analysis based on chlorophyta_odb10 (a total of 1519 gene set). 88.4% and 86.9% of genes were evaluated to be complete for the predicted genes in S1 and S931, respectively (**Table 1** and **Table S9**). The annotation of complete genes was higher in this study compared to *P. wickerhamii* strain ATCC 16529 (79.7%). Potentially, the greater volume of long-read sequencing data (4.5 Gb for S1 and 4.9 Gb for S931) led to more complete genome assemblies as compared to that of strain ATCC 16529 (2.2 Gb). The annotation of gene functions demonstrated that more than 95% of genes were annotated in both of the newly sequenced *P. wickerhamii* genomes (**Table S10**). The enrichment of Gene Ontology (GO) annotation showed a similar distribution of biological processes, cellular components, and molecular functions in strain S1 and S931, and these were the processes with the highest number of genes enriched in the global overview maps of Kyoto Encyclopedia of Genes and Genomes (KEGG) among all the pathways (**Figures S5–S8**).

**Table 1:** Genome statistics of two newly sequenced and one published *P. wickerhamii*.

*P. wickerhamii*	Strain S1	Strain S931	Strain ATCC 16529 (Bakula et al., 2021)
Assembly length (Mb)	17.57	17.45	16.70
Contig number	19	26	21
N50 contig (bp)	1,639,047	1,406,360	1,578,614
GC content (%)	64.21	64.45	64.16
Number of genes	5,694	5,704	6,081
Complete BUSCOs V5 (gene)	88.40%	86.90%	79.70%
Repetitive DNA in genome assembly (%)	3.11	2.49	2.25

### Phylogenetic Analysis

**Figure 2:**
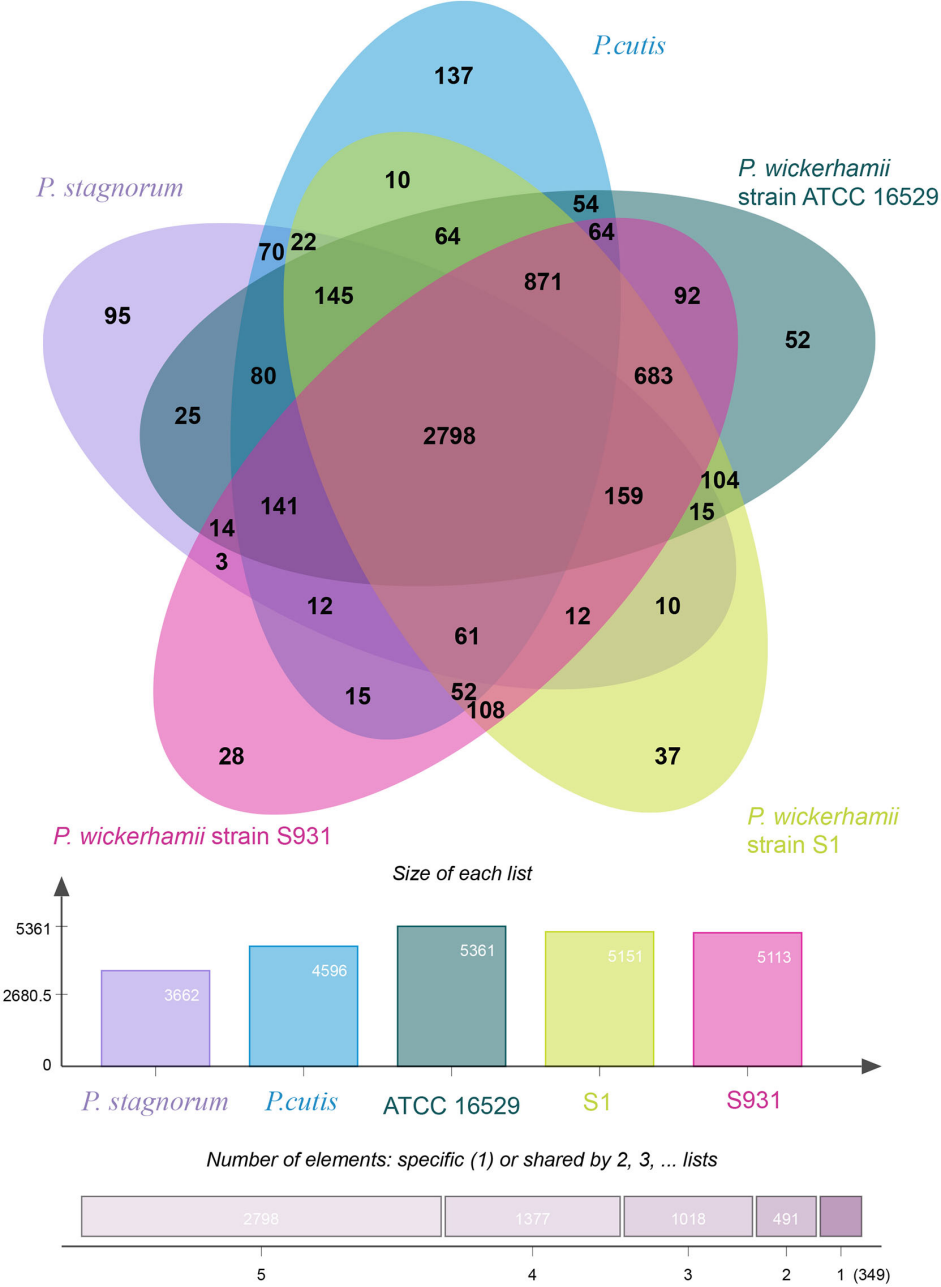
Distribution of gene families in *P. wickerhamii* strain S1, *P. wickerhamii* strain S931, *P. wickerhamii* strain ATCC 16529, *P. cutis, and P. stagnorum*. Homologous genes in the five species were clustered into gene families. Numbers indicate unique and shared gene families in each species.

**Figure 3:**
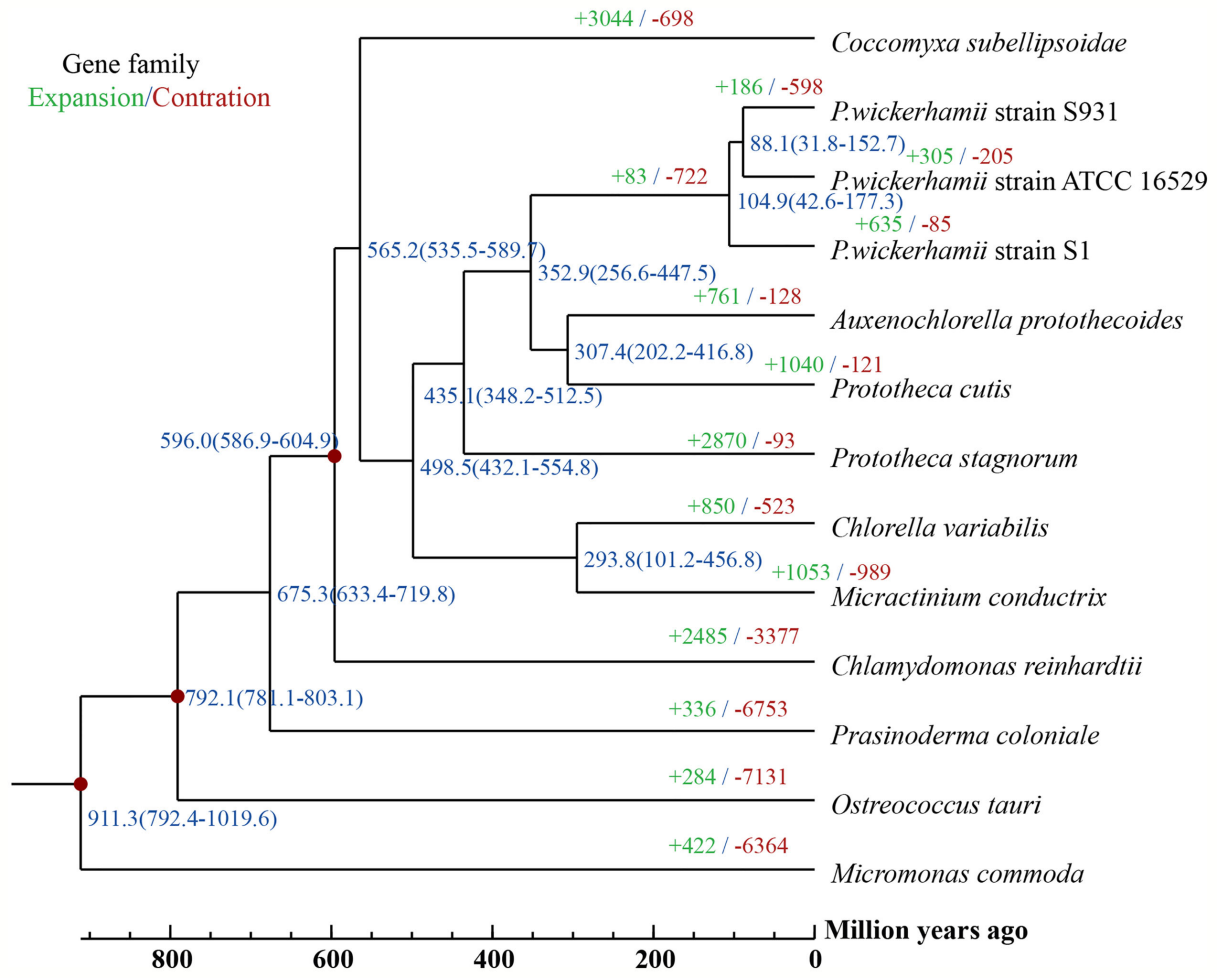
A Phylogenetic tree and gene family expansion and contraction among 13 species. Node labels represent node ages. Expansion/contraction of gene families is shown in green and red.

To gain insights into the evolution of *Prototheca*, the two newly sequenced genomes were compared with 11 other published, algal genomes including *Micromonas commoda*, *Ostreococcus tauri*, *Prasinoderma coloniale*, *Chlamydomonas reinhardtii*, *Micractinium conductrix*, *Chlorella variabilis*, *Auxenochlorella protothecoides*, *Coccomyxa subellipsoidae*, *Prototheca stagnorum*, *Prototheca cutis*, and *P. wickerhamii* strain ATCC 16529. *M. commoda* was used as the outgroup. The gene families were clustered for identifying the single-copy orthologous genes. The number of clustered gene families in these species ranged from 3,662 (*P. stagnorum*) to 8,012 (*C. reinhardtii*) (**Table S11**). The number of unique gene families in the five *Prototheca* strains was less than 10 (**Table S11**). A total of 2,798 gene families were shared between the five pathogenic *Prototheca* strains (**Figure 2**). 1,377, 1,018, 491, and 349 genes were shared in four, three, two, and one strain, respectively. Compared to the eight genomes of non-pathogenic algae, 494 gene families were unique to the five genomes of the pathogenic *Prototheca* strains. A total of 683 gene families were identified specific to *P. wickerhamii* in comparison to *P. stagnorum* and *P. cutis*. Finally, 737 single-copy orthologous gene families were shared by all 13 algal genomes. The phylogenetic tree implied that the genus of *Prototheca* forms a clade with *A. protothecoides* (**Figure 3**). The new sequenced *P. wickerhamii* strains S1 and S931 grouped in a single clade with strain ATCC 16529. Our analysis proposes that the genus of *Prototheca* diverged from *Chlorella* ~498.5 million years ago (Mya) and the species of *P. wickerhamii* separated from the common ancestor approximately 104.9 Mya (**Figure 3**). Following this divergence, 83 gene families showed expansion in species of *P. wickerhamii*, and 722 gene families showed contraction. Of the 83 expanded gene families, a total of 134 genes were enriched in several pathways within the KEGG analysis (**Table S12**). The genes were notably enriched in metabolic pathways, followed by biosynthesis of secondary metabolites and RNA transport (**Table S12**).

### Comparative Genomics and Putatively Involved in Pathogenicity With the PHI Database

For comparison of the three strains of *P. wickerhamii*, NUCmer v3.1 generated a total of 799 contig alignments between S1 and S931 averaging at 96.35% identity. 478 contig alignments were generated for S1 and ATCC 16529 with a sequence identity of 96.27%. The results of the sequence alignments showed highly conserved collinearity (**Figure S9**). Within the alignment results, a total of 94,236 SNPs were detected between strains S1 and S931 (**Table S13**). For the three sets of SNP comparisons in *P. wickerhamii*, 59.87%, 61.91%, and 58.60% were transitions in S1 vs. S931, S1 vs. ATCC 16529, and ATCC 16529 vs. S931, respectively, and the rate of transitions was higher than that of transversions (transition/transversion ratios ranged from 1.415 to 1.625) (**Table S13**). The SNP C/T was the most common (~30%) and A/T the least common (below 7.3%) substitution observed, which is different in comparison to findings in higher plant species (Lu et al., 2021).

**Figure 4:**
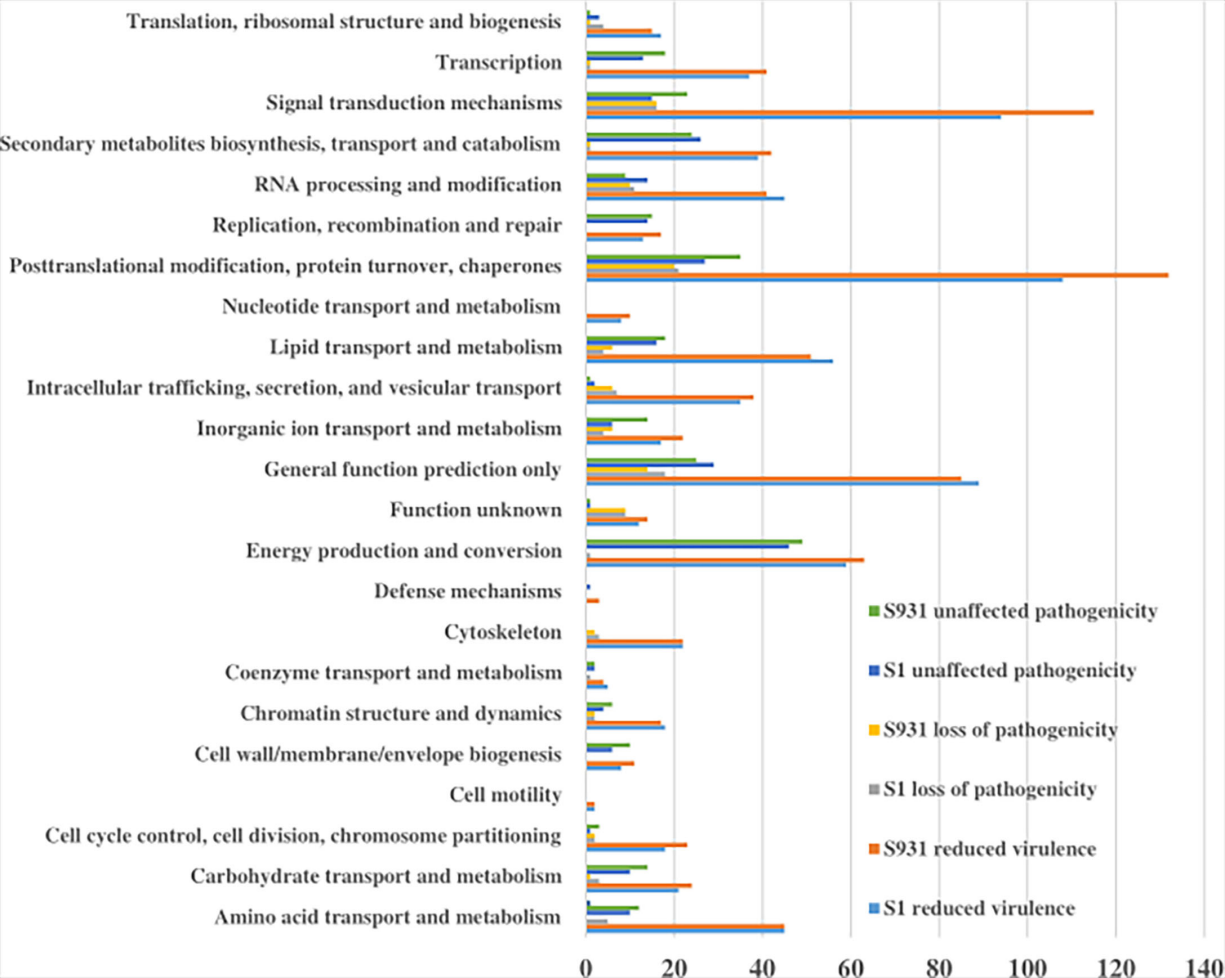
The KOG functional categories for “reduced virulence,” “unaffected pathogenicity,” and “loss of pathogenicity” in S1 and S931.

To evaluate potential pathogenicity-associated genes in the *Prototheca* genomes, genome-wide protein BLAST analysis against the Pathogen Host Interaction (PHI) database was performed. The abundances of protein-coding genes identified as orthologs to PHI genes were similar among the five *Prototheca* strains and ranged from 1,578 in *P. stagnorum*, 1,744 in *P. wickerhamii* strain S1, 1,775 in *P. wickerhamii* strain ATCC 16529, and 1,789 in *P. wickerhamii* strain S931 to 1,791 *P. cutis*. A total of 2,195 PHI-database genes were matched in all five *Prototheca* genomes: 1,088 genes were annotated with “reduced virulence,” 474 with “unaffected pathogenicity,” 175 with “loss of pathogenicity,” and 35 with both “reduced virulence” and “loss of pathogenicity” (**Supplementary Material 1**). A total of 23 KOG functional categories were annotated with “reduced virulence,” “unaffected pathogenicity,” and “loss of pathogenicity” in S1 and S931 (**Figure 4**). The most abundant KOG categories were posttranslational modification, protein turnover, chaperones, and signal transduction mechanisms. A total of 62 PHI genes were highly represented with ≥10 hits among all the five genomes. The pathogen gene encoding MoTup1 (PHI:4475), a general transcriptional repressor, resulted in the highest number of 499 total hits in the *Prototheca* genomes. MoTup1 is required for dimorphism and virulence in a fungal plant pathogen (Chen et al., 2015). The second most abundant hits were obtained for CaTUP1 (PHI:211) and tup1 (PHI:6806), which are associated with transcription factors and transcriptional repressors, respectively, suggesting that transcriptional regulation might be important for the *Prototheca* pathogenesis.

### Difference in Gene Expression Between Strain S1 and S931

To identify unique features in the pathogenicity of the two *P. wickerhamii* strains S1 and S931, comparative transcriptomic analysis of six samples (three biological replicates of each strain, S1 and S931) was performed. A total of 25.9 and 20.4 Gb high-quality data (with the adaptors removed and low-quality data filtered out) were obtained, and approximately 38.55% and 34.91% reads could be mapped to the genes of the *P. wickerhamii* genomes of S1 and S931, respectively. A total of 3,786 differentially expressed genes (DEGs) were identified in the two strains. 1,887 genes were upregulated, and 1,899 genes were downregulated in S1 compared to S931 (**Figure S10**). With integration of the PHI database and InterPro annotation, 413 upregulated and 426 downregulated genes could be annotated in the datasets. The highest upregulated gene (Maker00004366) was annotated with the function of an integral membrane component (GO:0016021) and as *satP* gene (PHI:9230) encoding a symporter involved in the pathogenicity of *Escherichia coli*. The highest downregulated gene (Maker00001286) was annotated with three GOs, namely, protein kinase activity (GO:0004672), ATP binding (GO:0005524), and protein phosphorylation (GO:0006468). This gene was also matched with several PHI genes including PHI:1239(FGSG_02399), PHI:1230(FGSG_06959), PHI:1186(Sc_Cdc15), PHI:1241(FGSG_00469), and PHI:1257 (FGSG_07121) associated with pathogenicity of *Fusarium graminearum*. These DEGs could play a role in the unique metabolic or pathogenic strategies of each strain during growth and infection.

## Discussion

The investigation of molecular epidemiology of *P. wickerhamii* is still limited. There were only a few studies including *P. wickerhamii* drug susceptibility tests and unrooted phylogenetic trees which were based on nuclear rDNA transcriptional units (Falcaro et al., 2021). *In vitro, P. wickerhamii* was generally susceptible to amphotericin and had variable susceptibility to triazoles (McMullan et al., 2016). The *Prototheca* CYP51/ERG11 and fungal CYP51/ERG11 were phylogenetically different from each other (Watanabe et al., 2021). Antifungal azoles had been used for the empirical treatment of protothecosis (Inoue et al., 2018). *In vitro*, the drug susceptibility tests of clinical and environmental isolates of *P. wickerhamii* to itraconazole (ITZ), voriconazole (VRZ), posaconazole (PCZ), and ravuconazole (RVZ) were different. RVZ was more effective than the other azoles against *Prototheca* species (Miura et al., 2020).

*Prototheca* genomes contain multiple copies of nuclear rDNA transcriptional units, each of which consists of an 18S small subunit (SSU) rDNA, 26S/28S large subunit (LSU) rDNA, ITS1, ITS2, and 5.8S rDNA (Hirose et al., 2013). The analysis of the *P. wickerhamii* 28S rRNA sequences revealed higher heterogeneity than cytb mitochondrial DNA sequences. A partial cytochrome b (CYTB) gene sequences had been used for identification of *P. wickerhamii* previously (Falcaro et al., 2021). The nucleotide sequences were heterogeneously different in rDNA copies of *P. wickerhamii* ITS, which might be useful for the intraspecies genotypic classification (Hirose et al., 2013). The *P. wickerhamii* ITS sequences could be grouped into four distinct clades (A to D). However, all of the strains examined only had the ITS of clades C and D (Hirose et al., 2013). The *P. wickerhamii* strains could be classified into at least two genotypes depending on ITS clades. Genotype 1 represented ITS clades A and B, and genotype 2 represented ITS clades C and D. The SSU rDNA of *P. wickerhamii* were very variable. Thus, the SSU-based intraspecies genotyping would be very difficult.

In this study, we compared two clinical strains of *P. wickerhamii* isolated in China for their rDNA by PCR and nucleotide sequencing. We obtained two *P. wickerhamii* genomes of strain S1 (17.57 Mb) and strain S931 (17.45 Mb). Assessing the assembly with two software tools, the Necat assembly resulted in a higher genome quality and completeness. The newly assembled genomes of both strains were nearly 1 Mb larger than the previously published genome of the type strain ATCC 16529 (16.7 Mb) (Bakula et al., 2021). Also, percentages of repetitive elements were slightly higher than for ATCC 16529 (Bakula et al., 2021). The greater abundance of repetitive elements might explain the higher assembled genome size with a higher level of genome quality and completeness. The small assembled genome size and low content of repetitive elements suggest that the *P. wickerhamii* genome is one of the smallest and most compact microalgal genomes.

The genus of *Prototheca* is the only algae to be involved in a series of clinically relevant opportunistic infections in humans and animals (Kwiecinski, 2015). Most human pathogenic cases are caused by *P. wickerhamii*, which suggests that this species may have undergone an evolutionary specification process. The pathogenicity may come from the horizontal gene transfer or new evolutionary functions acquired. So far, genomic information on *Prototheca* has been limited and previous analysis of plastid genomes suggested that *P. wickerhamii* and its closest photosynthetic relative *A. protothecoides* diverged 6–20 Mya (Yan et al., 2015). In contrast, our analysis based on the nuclear genomes suggests an even earlier divergence from a common ancestor at around 350 Mya. Phylogenetically, *A. protothecoides* is however still placed within the clade of *Prototheca*, which opens up interesting questions about the loss of photosynthesis and the evolution of protothecosis in *Prototheca*.

The three strains of *P. wickerhamii* were classified in the same clade. Although the collinearity of sequence alignment showed high sequence consistency between the strains, the detected SNPs may be used as a resource for the identification.

A comparison of the *Prototheca* genomes against the Pathogen Host Interaction (PHI) identified potential pathogenicity-associated genes that were enriched highly in the KOG functional categories of posttranslational modification, protein turnover, chaperones, and signal transduction mechanisms. These functional genes included those involved in the ATP-binding cassette (ABC) transporter, which could be linked to nutrient uptake, drug resistance, or bacterial pathogenesis. Three DnaJ (PHI:10485, PHI:4733, and PHI:6986) genes were annotated with heat shock protein and enriched in the PI3K 383 and JNK signaling pathways. These results were similar with the previous report in the *P. bovis*-induced infections (Murugaiyan et al., 2016). The pathogen gene encoding MoTup1 resulted in the highest abundance of hits in *Prototheca* and has important regulatory roles in the growth and development of fungi (Chen et al., 2015). These results agree with a previous study indicating that putative pathogenicity genes in *Prototheca* are similar to those in fungi (Bakula et al., 2021). Also, annotation of the high DEGs in S1 and S931 suggests similarities to fungal pathogenicity. The two types of *P. wickerhamii* strains showed different culture phenotypes. The sporophyte of strain S1 (mucoid colony) was smaller than that of strain S931 (rough colony). The metabolic or pathogenic strategies may be caused by these DEGs.

## Conclusion

In this study, we assembled the high-quality mitochondrial (mtDNA), plastid (ptDNA), and nuclear genomes of two *P. wickerhamii* strains. Both mtDNA and ptDNA genome sequences were assembled in one circular contig. The nuclear genomes of strain S1 (17.57 Mb with 19 contigs) and S931 (17.45 Mb with 26 contigs) were nearly at the chromosome level. The comparative genomics and evolutionary analysis showed that the genus of *Prototheca* was closely related to *A. protothecoides* and diverged from *Chlorella* 500 Mya. The species-specific differences in the genetics, pathogenicity, and differentially expressed genes of the *P. wickerhamii* strains have been discovered. The high-quality genomes provide a valuable reference for the evolutionary and pathogenicity studies of *Prototheca* and provide genomic resources for the diagnosis of protothecosis.

## Data Availability Statement

The datasets presented in this study can be found in online repositories. The names of the repository/repositories and accession number(s) can be found in the article/**Supplementary Material**.

## Ethics Statement

The studies involving human participants were reviewed and approved by the ethics committee of Shanghai East Hospital, Tongji University School of Medicine (No. 2020-163). Written informed consent for participation was not required for this study in accordance with the national legislation and the institutional requirements.

## Author Contributions

WW and ES: conception and design. JG, JJ, and LW: whole-genome sequencing and data analysis. LX, HL, ZZ, and ES: data analysis and interpretation. All authors: collection and assembly of data, manuscript writing, and final approval of manuscript. All authors contributed to the article and approved the submitted version.

## Funding

This work was supported by the National Natural Science Foundation [grant number 81971990], Key Discipline of Public Health in Shanghai [grant number GWV-10.1-XK04], Excellent Technology Leader in Shanghai [grant number 20XD1434500], and Novo Nordisk Foundation [grant number NNF20OC0064249].

## Conflict of Interest

The authors declare that the research was conducted in the absence of any commercial or financial relationships that could be construed as a potential conflict of interest.

## Publisher’s Note

All claims expressed in this article are solely those of the authors and do not necessarily represent those of their affiliated organizations, or those of the publisher, the editors and the reviewers. Any product that may be evaluated in this article, or claim that may be made by its manufacturer, is not guaranteed or endorsed by the publisher.
