# Supplementary material for: Genome Sequences of Two Strains of Prototheca wickerhamii Provide Insight Into the Protothecosis Evolution
Source: Front Cell Infect Microbiol. 2022 Feb 2;12:797017. doi: 10.3389/fcimb.2022.797017 (PMC8847788; doi:10.3389/fcimb.2022.797017)
Supplement: Supplementary file 1 [file DataSheet_1.docx]

**Genome sequences of two strains of *Prototheca wickerhamii* provide insight into the protothecosis evolution**

**Jian Guo ^1#^, Jianbo Jian^2#^, Lili Wang^1#^, Lijuan Xiong^3^, Huiping Lin^1^, Ziyi Zhou^1^, Eva C. Sonnenschein ^2*^, Wenjuan Wu^1*^**

Supplementary materials

Outline

[Supplementary section: Plastid, mitochondrion and nuclear genomes assemblies 2](#_Toc89714099)

[Figure S1 The cultured phenotype of *P. wickerhamii* strain S1 and S931. 3](#_Toc89714100)

[Figure S2 Genome characteristics estimation of *P. wickerhamii* strain S1 using GenomeScope. 4](#_Toc89714101)

[Figure S3 Genome characteristics estimation of *P. wickerhamii* strain S931 using GenomeScope. 5](#_Toc89714102)

[Figure S4 Circular map of genomic features of mitochondria and plastid of *P. wickerhamii* genomes. 6](#_Toc89714103)

[Figure S5 The Gene Ontology (GO) enrichment annotation of *P.wickerhamii* strain S1 7](#_Toc89714104)

[Figure S6 Kyoto Encyclopedia of Genes and Genomes (KEGG) enrichment annotation of *P. wickerhamii* strain S1 8](#_Toc89714105)

[Figure S7 The Gene Ontology (GO) enrichment annotation of *P. wickerhamii* strain S931 9](#_Toc89714106)

[Figure S8 Kyoto Encyclopedia of Genes and Genomes (KEGG) enrichment annotation of *P. wickerhamii* strain S931 10](#_Toc89714107)

[Figure S9 Genomic comparison of the three *P. wickerhamii* strains S1, S931, and ATCC 16529. 10](#_Toc89714108)

[Figure S10 Differences in gene expression between *P. wickerhamii* strains S1 and S931. 11](#_Toc89714109)

[Table S1 Sequencing data statistics of two types of *P. wickerhamii* genomes with Illumina platform. 11](#_Toc89714110)

[Table S2 Sequencing data statistics of two types of *P. wickerhamii* genomes with Nanopore platform. 11](#_Toc89714111)

[Table S3. The assembly statistics of *P. wickerhamii* genomes. 12](#_Toc89714112)

[Table S4. BUSCO evaluation of *P. wickerhamii* genome assembly. 12](#_Toc89714113)

[Table S5 General features of the mitochondrion and plastid genomes of *P. wickerhamii* 13](#_Toc89714114)

[Table S6 The statistics of *Prototheca wickerhamii* strain S1 assembled nuclear genome. 13](#_Toc89714115)

[Table S7. The statistics of *Prototheca wickerhamii* strain S931 assembled nuclear genome. 13](#_Toc89714116)

[Table S8 Repeat content in the assembled nuclear genomes. 14](#_Toc89714117)

[Table S9 BUSCO analysis result for different *P. wickerhamii* including the genome and gene evaluation. 15](#_Toc89714118)

[Table S10 Gene function annotation 15](#_Toc89714119)

[Table S11 Gene family statistics 16](#_Toc89714120)

[Table S12 KEGG pathway enrichment annotation in expansion of *P.wickerhamii* species 16](#_Toc89714121)

[Table S13 The SNP information in *P.wickerhamii* genomes. 23](#_Toc89714122)

# Supplementary section: Plastid, mitochondrion and nuclear genomes assemblies

The clean Nanopore long reads were used to obtain two primary genome sequences with Canu and Necat, respectively. The assembled genomes were self-corrected and polished with Nanopore long reads and Illumina short reads. Finally, the assembled S1 genome size is 19,936,577 bp with Contig N50=1,648,552 and 84 contigs using the Canu-based pipeline. A total of 17,674,137 bp were assembled with Contig N50=1,639,047 and 21 contigs using Necat. Similar assembly results were generated for S931: the assembled genome size is respectively 19,980,883 bp (Contig N50=1,082,058 bp; 79 Contigs) with Canu and 17,651,439 bp (Contig N50=1,406,360 bp; 31 Contigs) with Necat (Table S3). The GC content of all the assembled genome is approximately 64 %, being slightly higher with Necat than Canu (Table S3). The mean length of the assembled genome in Necat is significantly longer than that of Canu revealing the better assembly in Necat. The assembled genome size in Canu is about 20 Mb, which is larger than the estimated genome size (18.65 Mb and 17.97 Mb) suggesting redundant assembly. The assembled genome sequences were further evaluated with BUSCO. The complete BUSCOs (C) of the genomes were 87.1% and 88.8% for S1 and S931, respectively. However, the complete and duplicated BUSCOs (D) are 7.6% in S1 and 8.2% in S931 for the Canu assembly (Table S4). The complete and duplicated BUSCOs (D) are 1.3 % in S1 and 2.4 % in S931 for the Necat assembly (Table S4). The significantly higher number of duplicated genes in Canu than in Necat means that it is true that there are redundant sequences in Canu-based assembly. Therefore, the Necat-based genome was used for downstream analyses.

According to the previous reported mitochondrial (mtDNA) and plastid (ptDNA) genomes of the *P. wickerhamii* type strain ATCC 16529 (Bakula et al., 2021), the assembled genome sequences were blasted to identify the mtDNA and ptDNA in S1 and S931. In S1, only contig 19 (52,657) and contig 21 (47,502 bp) could be blasted with >1 kb with ATCC 16529 mtDNA (MN794237.1, 53.8 kb) and ATCC 16529 ptDNA (MN794236.1, 48 kb kb), respectively. A similar result was found for S931, with the contigs matching ATCC 16529 mtDNA and ptDNA being 57,870 bp and 47,464 bp in length (Figure 2). The number of CDSs, rRNAs, tRNAs in the mitochondrion and plastid genomes were similar across the three *P. wickerhamii* strains. The GC content of the mitochondrion genomes ranges from 25.63% to 25.81% and the GC content of the plastid genomes is about 28.2% (Table S5).

# Figure S1 The cultured phenotype of *P. wickerhamii* strain S1 and S931.

Left：*Prototheca* *wickerhamii* S931 (rough colony)；

Right：*Prototheca* *wickerhamii* S1 (mucoid colony)

# Figure S2 Genome characteristics estimation of *P. wickerhamii* strain S1 using GenomeScope.


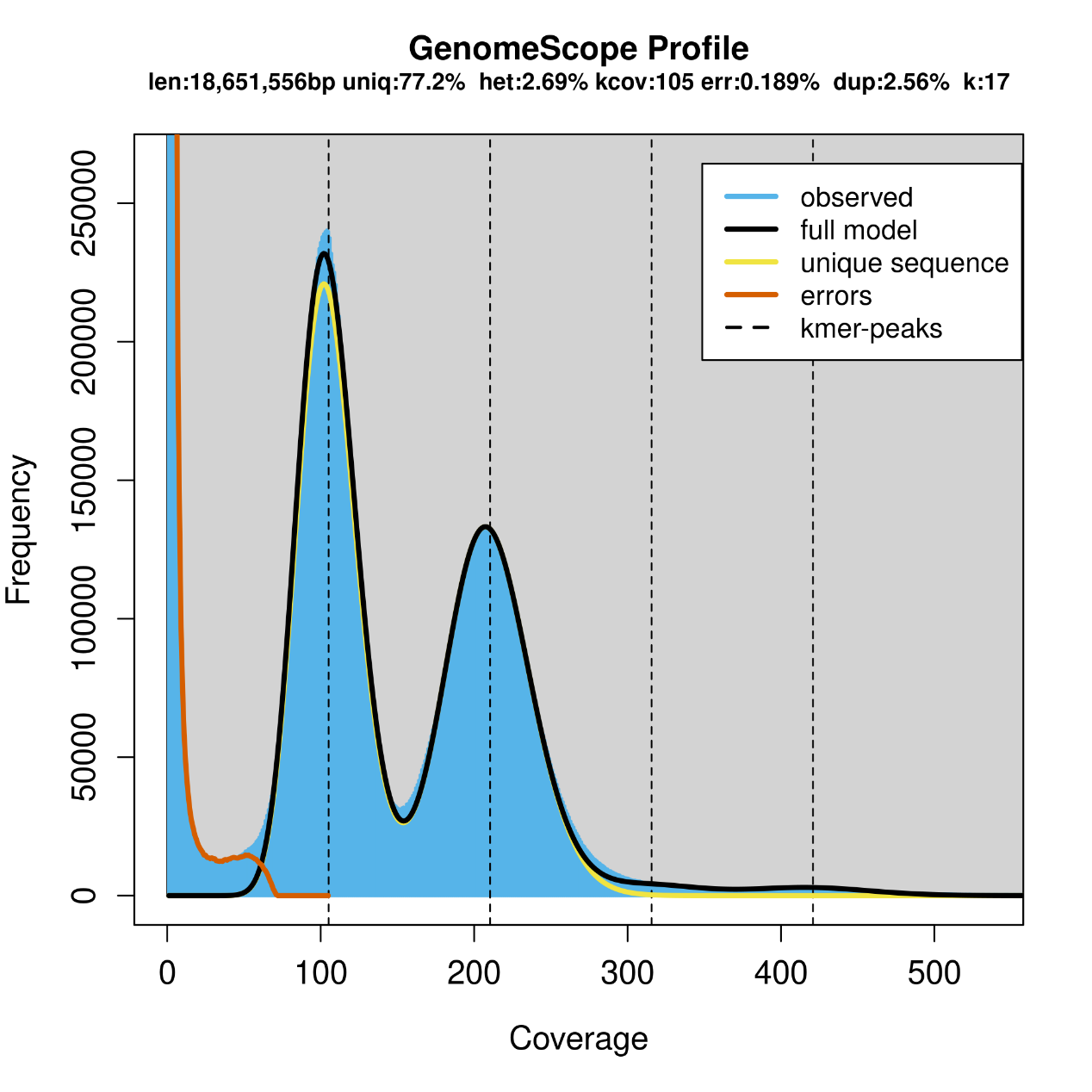


**Figure S2 Genome characteristics estimation of *P. wickerhamii* strain S1 using GenomeScope.** X axes is the coverage (X), y axes is the frequency of 17-mer.

Note: het: heterozygosity; kcov: kmer mean peak; uniq: non-repetitive kmer; observed: the kmer actual distribution by jellyfish analysis; full model: theoretical kmer distribution; unique sequence: non-repetitive kmer; errors: wrong kmer, usually the lower kmer；kmer-peaks: the position of the kmer peak.

# Figure S3 Genome characteristics estimation of *P. wickerhamii* strain S931 using GenomeScope.


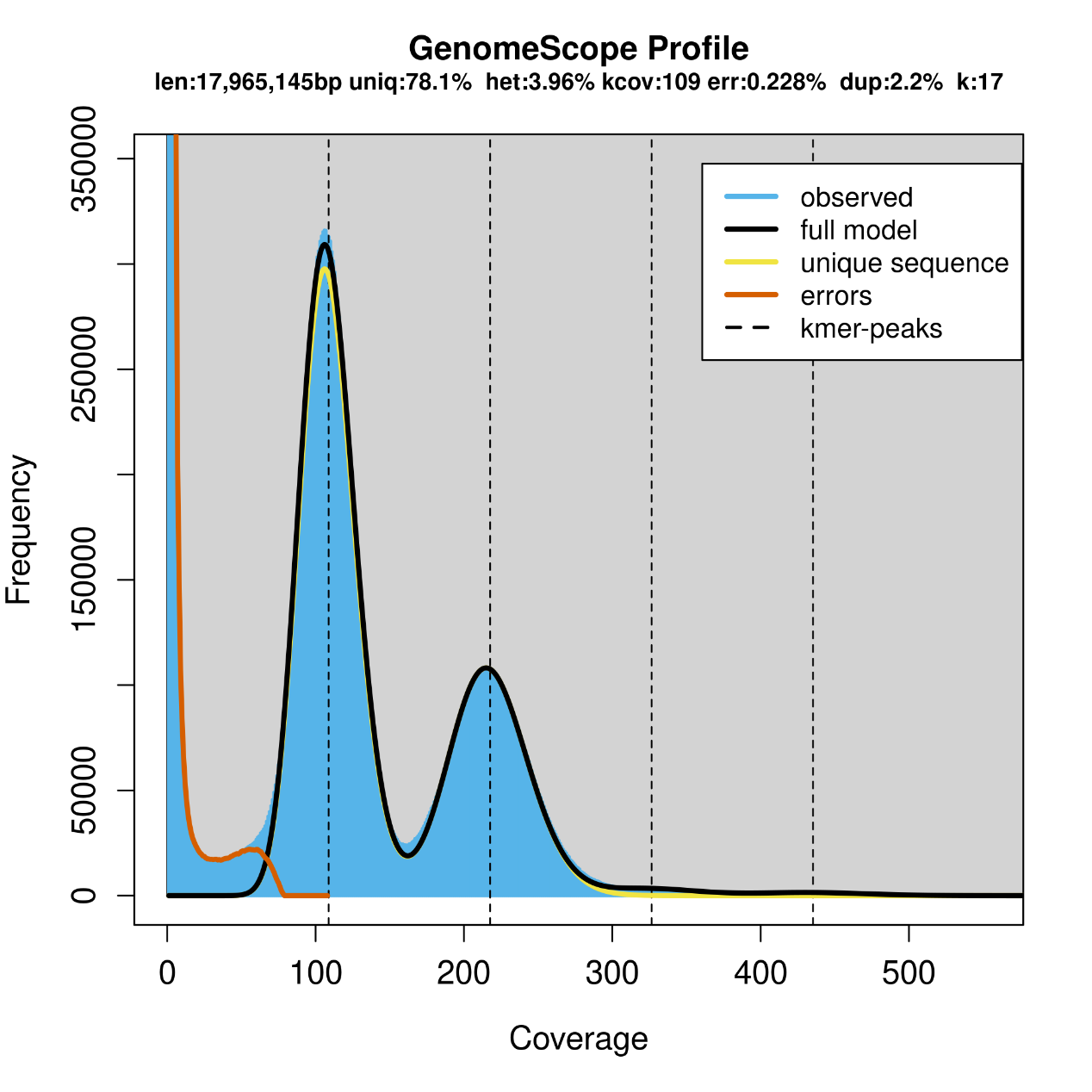


**Figure S3 Genome characteristics estimation of *P. wickerhamii* strain S1 using GenomeScope.** X axes is the coverage (X), y axes is the frequency of 17-mer.

Note: het: heterozygosity; kcov: kmer mean peak; uniq: non-repetitive kmer; observed: the kmer actual distribution by jellyfish analysis; full model: theoretical kmer distribution; unique sequence: non-repetitive kmer; errors: wrong kmer, usually the lower kmer；kmer-peaks: the position of the kmer peak.

# Figure S4 Circular map of genomic features of mitochondria and plastid of *P. wickerhamii* genomes.


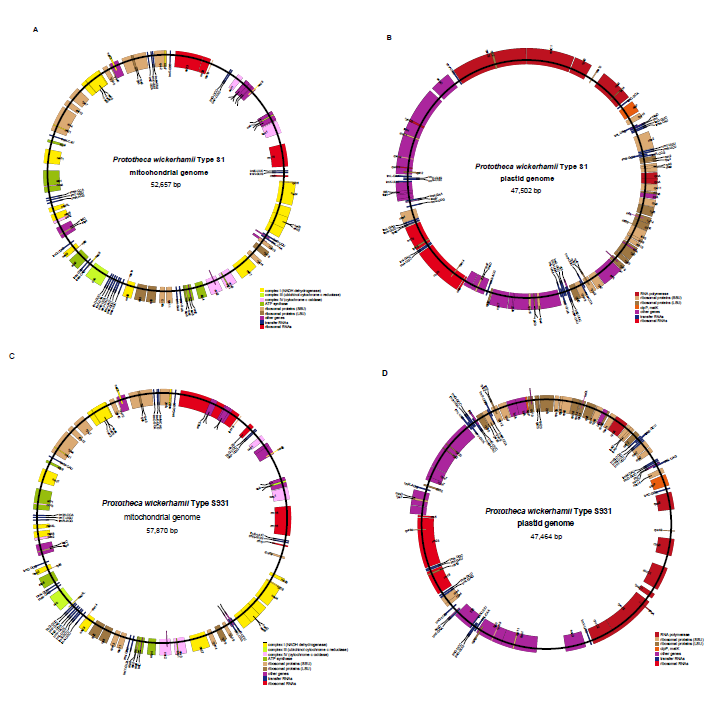


**Figure S4 Circular map of genomic features of mitochondria and plastid of *P. wickerhamii* genomes.** A: Strain S1 mitochondrial genome. B: Strain S1 plastid genome. C: Strain S931 mitochondrial genome. D: Strain S931 plastid genome.

# Figure S5 The Gene Ontology (GO) enrichment annotation of *P.wickerhamii* strain S1


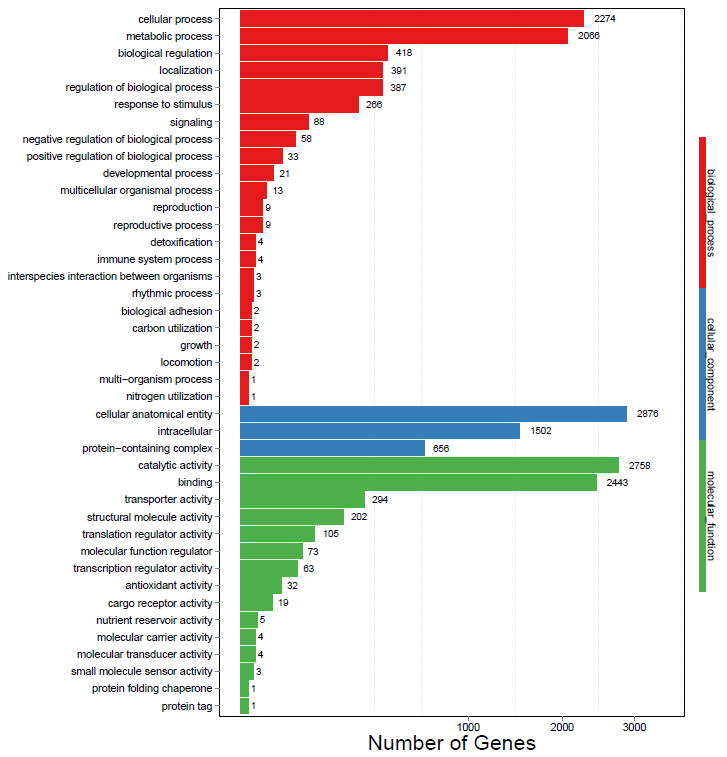


# Figure S6 Kyoto Encyclopedia of Genes and Genomes (KEGG) enrichment annotation of *P. wickerhamii* strain S1


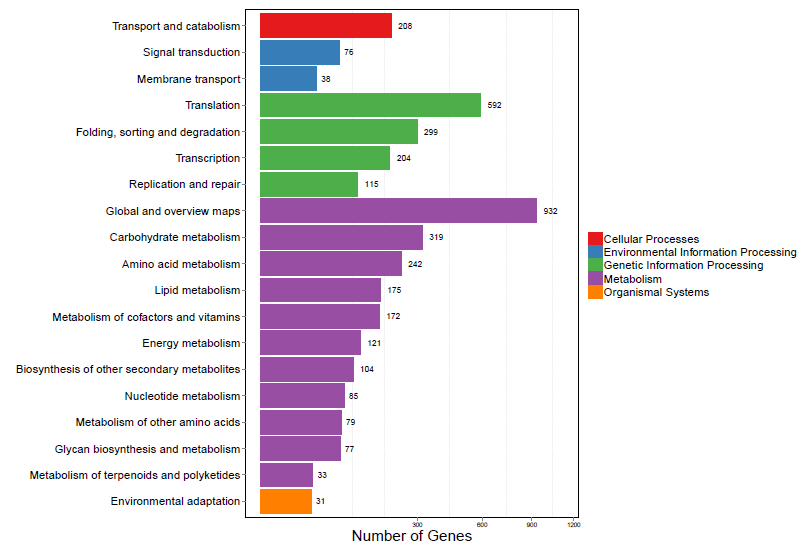


# Figure S7 The Gene Ontology (GO) enrichment annotation of *P. wickerhamii* strain S931


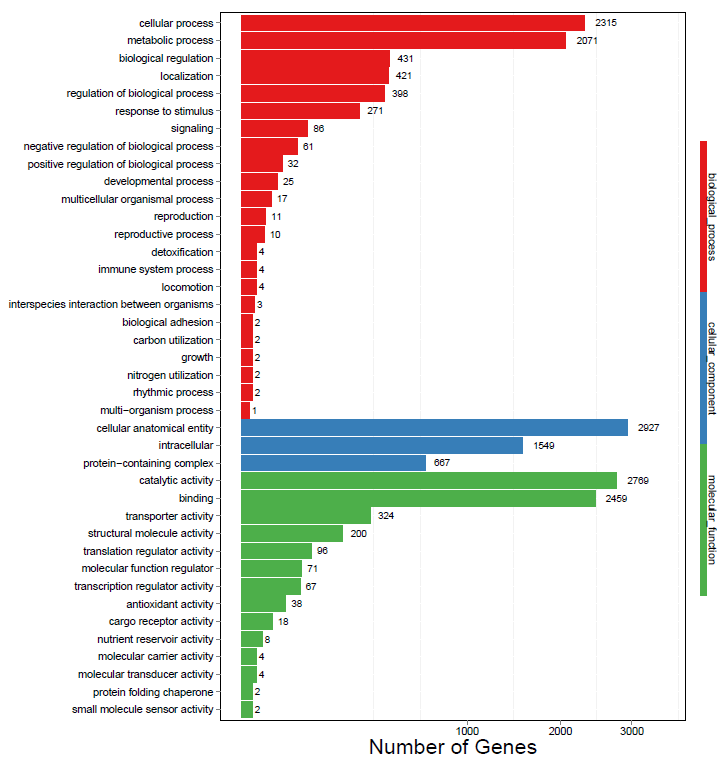


# Figure S8 Kyoto Encyclopedia of Genes and Genomes (KEGG) enrichment annotation of *P. wickerhamii* strain S931


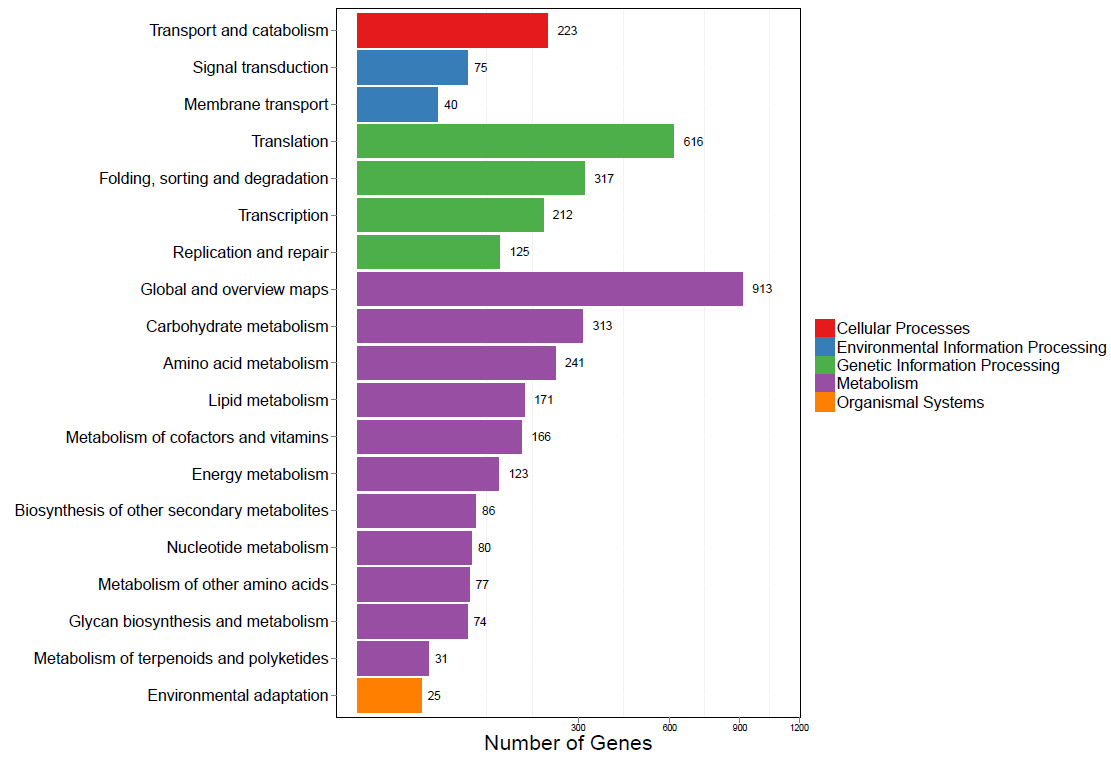


# Figure S9 Genomic comparison of the three *P. wickerhamii* strains S1, S931, and ATCC 16529.


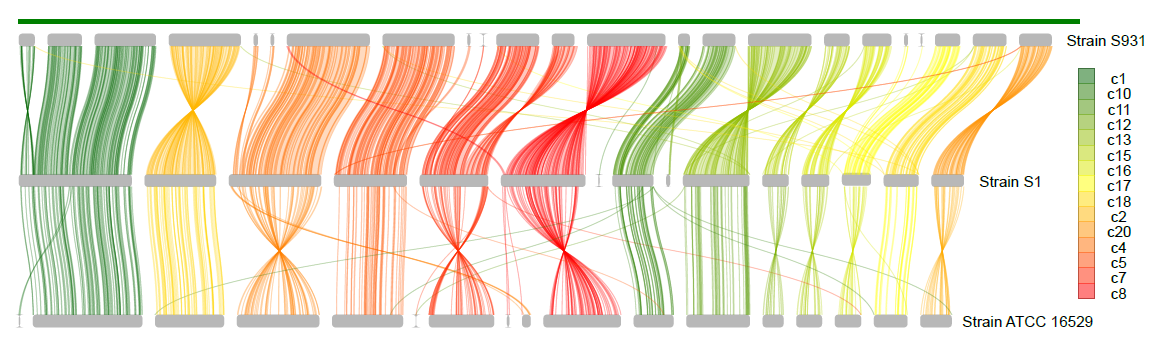


# Figure S10 Differences in gene expression between *P. wickerhamii* strains S1 and S931.


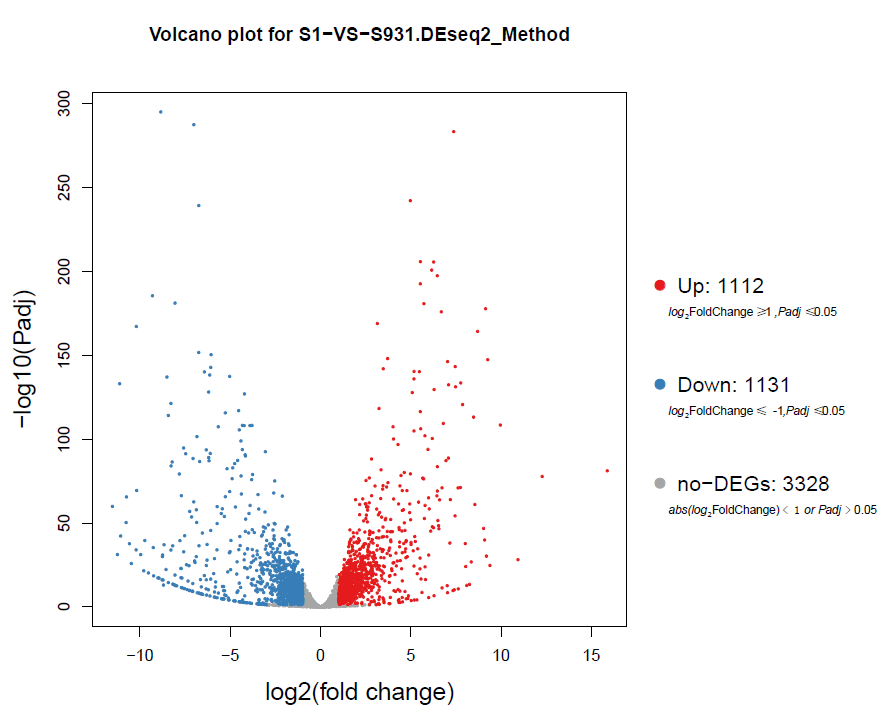


# Table S1 Sequencing data statistics of two types of *P. wickerhamii* genomes with Illumina platform.

Table S1 Sequencing data statistics of two types of *P.wickerhamii* genomes with Illumina platform.

| **Sample** | **Clean Reads** | **Clean Data (bp)** | **Q20 (%)** | **Q30 (%)** | **GC (%)** |
| --- | --- | --- | --- | --- | --- |
| S1 | 30,294,638 | 4,538,051,122 | 98.16 | 95.02 | 58.30 |
| S931 | 30,400,388 | 4,551,834,050 | 98.09 | 94.92 | 58.99 |

# Table S2 Sequencing data statistics of two types of *P. wickerhamii* genomes with Nanopore platform.

Table S2 Sequencing data statistics of two types of *P.wickerhamii* genomes with Nanopore platform.

| **Sample** | **Total reads** | **Total bases (bp)** | **Average length (bp)** | **Max length (bp)** |
| --- | --- | --- | --- | --- |
| S1 raw data | 1,328,625 | 4,945,169,571 | 3,722 | 153,066 |
| S1 clean data | 551,989 | 4,545,371,048 | 8,235 | 153,066 |
| S931 raw data | 681,088 | 4,984,644,056 | 7,319 | 149,724 |
| S931 clean data | 542,630 | 4,902,398,079 | 9,035 | 149,724 |

# Table S3. The assembly statistics of *P. wickerhamii* genomes.

Table S3. The assembly statistics of *P.wickerhamii* genomes**.**

|  | **S1** | | **S931** | |
| --- | --- | --- | --- | --- |
|  | **Canu** | **Necat** | **Canu** | **Necat** |
| Assembled genome size (bp) | 19,936,577 | 17,674,137 | 19,980,883 | 17,651,439 |
| Contig Number | 84 | 21 | 79 | 31 |
| Contig N50 | 1,648,552 | 1,639,047 | 1,082,058 | 1,406,360 |
| Max length | 2,551,560 | 2,552,045 | 2,083,189 | 1,845,983 |
| Mean length | 237,340 | 841,625 | 252,922 | 569,401 |
| GC% | 63.54% | 64.00% | 63.73% | 64.04% |

Note: the assembled genomes contained the nuclear genomes, mitochondrion and plastid genomes.

# Table S4. BUSCO evaluation of *P. wickerhamii* genome assembly.

Table S4 BUSCO evaluation of *P.wickerhamii* genome assembly

| **Type** | **S1_Necat** | | **S1_Canu** | | **S931_Necat** | | **S931_Canu** | |
| --- | --- | --- | --- | --- | --- | --- | --- | --- |
|  | **Number** | **Percentage (%)** | **Number** | **Percentage (%)** | **Number** | **Percentage (%)** | **Number** | **Percentage (%)** |
| Complete BUSCOs (C) | 1348 | 88.8 | 1347 | 88.7 | 1336 | 87.1 | 1336 | 87.9 |
| Complete and single-copy BUSCOs (S) | 1329 | 87.5 | 1232 | 81.1 | 1211 | 84.7 | 1211 | 79.7 |
| Complete and duplicated BUSCOs (D) | 19 | 1.3 | 115 | 7.6 | 125 | 2.4 | 125 | 8.2 |
| Fragmented BUSCOs (F) | 11 | 0.7 | 10 | 0.7 | 37 | 1.2 | 14 | 0.9 |
| Missing BUSCOs (M) | 160 | 10.5 | 162 | 10.6 | 169 | 11.7 | 169 | 11.2 |
| Total BUSCO groups searched | 1519 | 100 | 1519 | 100 | 1519 | 100 | 1519 | 100 |

# Table S5 General features of the mitochondrion and plastid genomes of *P. wickerhamii*

| **Genome** | **Type strain** | **Size (bp)** | **CDS** | **rRNA** | **tRNA** | **GC %** |
| --- | --- | --- | --- | --- | --- | --- |
| Mitochondrion | S931 | 57,870 | 42 | 5 | 26 | 25.66 |
|  | S1 | 52,657 | 41 | 4 | 26 | 25.63 |
|  | ATCC 16529 | 53,822 | 38 | 3 | 27 | 25.81 |
| Plastid | S931 | 47,464 | 36 | 3 | 29 | 28.26 |
|  | S1 | 47,502 | 36 | 3 | 29 | 28.22 |
|  | ATCC 16529 | 47,997 | 35 | 3 | 30 | 28.20 |

# Table S6 The statistics of *Prototheca wickerhamii* strain S1 assembled nuclear genome.

| S1 | Length | GC | GC content (%) |
| --- | --- | --- | --- |
| Contig1 | 2,552,045 | 1,648,168 | 64.58% |
| Contig4 | 2,086,090 | 1,348,001 | 64.62% |
| Contig8 | 1,901,190 | 1,230,371 | 64.72% |
| Contig5 | 1,660,611 | 1,075,347 | 64.76% |
| Contig2 | 1,639,047 | 1,044,207 | 63.71% |
| Contig7 | 1,560,028 | 998,295 | 63.99% |
| Contig13 | 1,491,610 | 957,776 | 64.21% |
| Contig11 | 928,735 | 589,869 | 63.51% |
| Contig18 | 793,799 | 504,738 | 63.59% |
| Contig20 | 744,247 | 476,778 | 64.06% |
| Contig17 | 659,570 | 426,089 | 64.60% |
| Contig16 | 648,131 | 410,554 | 63.34% |
| Contig15 | 601,769 | 381,193 | 63.35% |
| Contig12 | 110,109 | 66,155 | 60.08% |
| Contig6 | 62,121 | 44,436 | 71.53% |
| Contig14 | 59,188 | 33,109 | 55.94% |
| Contig3 | 43,908 | 28,574 | 65.08% |
| Contig10 | 30,119 | 19,390 | 64.38% |
| Contig9 | 1,661 | 1,203 | 72.43% |
| **Total** | **17,573,978** |  | **64.21%** |

# Table S7. The statistics of *Prototheca wickerhamii* strain S931 assembled nuclear genome.

Table S7. The statistics of *Prototheca wickerhamii* strain S931 assembled nuclear genome.

| **S931** | **Length** | **GC** | **GC content (%)** |
| --- | --- | --- | --- |
| Contig4 | 1,845,983 | 1,198,075 | 64.90% |
| Contig2 | 1,774,155 | 1,153,826 | 65.04% |
| Contig13 | 1,614,017 | 1,044,597 | 64.72% |
| Contig1 | 1,611,696 | 1,031,548 | 64.00% |
| Contig5 | 1,423,260 | 921,827 | 64.77% |
| Contig24 | 1,406,360 | 909,518 | 64.67% |
| Contig17 | 1,013,720 | 650,845 | 64.20% |
| Contig23 | 849,189 | 553,389 | 65.17% |
| Contig25 | 785,291 | 501,028 | 63.80% |
| Contig26 | 767,406 | 492,227 | 64.14% |
| Contig21 | 737,940 | 475,517 | 64.44% |
| Contig30 | 693,932 | 437,042 | 62.98% |
| Contig8 | 621,843 | 394,370 | 63.42% |
| Contig16 | 600,272 | 395,626 | 65.91% |
| Contig19 | 569,196 | 365,004 | 64.13% |
| Contig20 | 360,942 | 230,944 | 63.98% |
| Contig22 | 266,955 | 165,438 | 61.97% |
| Contig3 | 138,771 | 91,063 | 65.62% |
| Contig15 | 109,235 | 69,332 | 63.47% |
| Contig31 | 78,238 | 51,109 | 65.33% |
| Contig9 | 76,185 | 49,359 | 64.79% |
| Contig6 | 61,514 | 36,597 | 59.49% |
| Contig12 | 23,214 | 15,006 | 64.64% |
| Contig18 | 13,174 | 8,937 | 67.84% |
| Contig14 | 5,433 | 3,095 | 56.97% |
| Contig7 | 5,268 | 3,423 | 64.98% |
| **Total** | **17,453,189** |  | **64.45%** |

# Table S8 Repeat content in the assembled nuclear genomes.

Table S8 Repeat content in the assembled nuclear genomes.

| **Type** | **S1** | | **S931** | |
| --- | --- | --- | --- | --- |
|  | **Repeat Size** | **% of genome** | **Repeat Size** | **% of genome** |
| **Trf** | 432,804 | 2.46 | 331,641 | 1.90 |
| **Repeatmasker** | 96,782 | 0.55 | 74,804 | 0.43 |
| **Proteinmask** | 70,688 | 0.40 | 57,776 | 0.33 |
| **De novo** | 2,016 | 0.01 | 4,215 | 0.02 |
| **Total** | 546,360 | 3.11 | 434,225 | 2.49 |

Note: Repbase TEs: the result of *RepeatMasker* based on Repbase; TE proteins: the result of *RepeatProteinMask* based on Repbase; *De novo*: Result of *RepeatMasker* by using library predicted through *De novo*; Total: combine the results of Repbase TEs, TE proteins and *De novo* with removing the over-lap.

# Table S9 BUSCO analysis result for different *P. wickerhamii* including the genome and gene evaluation.

Table S9 BUSCO analysis result for *P. wickerhamii* including the genome and gene evaluation.

| ***P. wickerhamii*** | **Type** | **Complete BUSCOs (C)** | **Complete and single-copy BUSCOs (S)** | **Complete and duplicated BUSCOs (D)** | **Fragmented BUSCOs (F)** | **Missing BUSCOs (M)** | **Total BUSCO groups searched** |
| --- | --- | --- | --- | --- | --- | --- | --- |
| Strain S1 | Genome BUSCO (number) | 1314 | 1297 | 17 | 25 | 180 | 1519 |
|  | Genome BUSCO (percentage %) | 86.5 | 85.4 | 1.1 | 1.6 | 11.9 | 100 |
|  | Gene BUSCO (number) | 1343 | 1325 | 18 | 18 | 171 | 1519 |
|  | Gene BUSCO (percentage %) | 88.4 | 87.2 | 1.2 | 0.3 | 11.3 | 100 |
| Strain S931 | Genome BUSCO (number) | 1289 | 1258 | 31 | 29 | 201 | 1519 |
|  | Genome BUSCO (percentage %) | 8.8 | 8.8 | 2 | 1.9 | 13.3 | 100 |
|  | Gene BUSCO (number) | 1319 | 1277 | 42 | 14 | 186 | 1519 |
|  | Gene BUSCO (percentage %) | 86.9 | 84.1 | 2.8 | 0.9 | 12.2 | 100 |
| Strain ATCC 16529 | Genome BUSCO (number) | 1260 | 1255 | 5 | 44 | 215 | 1519 |
|  | Genome BUSCO (percentage %) | 82.9 | 82.6 | 0.3 | 2.9 | 14.2 | 100 |
|  | Gene BUSCO (number) | 1211 | 1199 | 12 | 54 | 254 | 1519 |
|  | Gene BUSCO (percentage %) | 79.7 | 78.9 | 0.8 | 3.6 | 16.7 | 100 |

# Table S10 Gene function annotation

Table S10 Gene function annotation in *P. wickerhamii* strain S1 and S931.

| ***P. wickerhamii*** | **Values** | **Total** | **Nr** | **Swissprot** | **KEGG** | **KOG** | **TrEMBL** | **GO** | **Interpro** | **Overall** |
| --- | --- | --- | --- | --- | --- | --- | --- | --- | --- | --- |
| Strain S1 | Number | 5,694 | 5,508 | 4,093 | 5,050 | 3,856 | 5,259 | 4,566 | 4,756 | 5,512 |
|  | Percentage | 100% | 96.73% | 71.88% | 88.69% | 70.72% | 92.36% | 80.19% | 83.53% | 96.80% |
| Strain S931 | Number | 5,704 | 5,548 | 4,108 | 5,067 | 3,881 | 5,307 | 4,622 | 4,313 | 5,554 |
|  | Percentage | 100% | 97.27% | 72.02% | 88.83% | 68.04% | 93.04% | 81.03% | 75.61% | 97.37% |

# Table S11 Gene family statistics

Table S11. Statistics of gene families in different species

| **Species** | **Genes number** | **Genes in families** | **Unclustered genes** | **Family number** | **Unique families** | **Average genes per family** |
| --- | --- | --- | --- | --- | --- | --- |
| *P. stagnorum* | 4,050 | 3,931 | 119 | 3,662 | 2 | 1.07 |
| *P. cutis* | 5,038 | 4,915 | 123 | 4,596 | 2 | 1.07 |
| *M. conductrix* | 10,041 | 9,018 | 1,023 | 6,875 | 226 | 1.31 |
| *C. subellipsoidae* | 9,815 | 7,781 | 2,034 | 6,253 | 223 | 1.24 |
| *C. variabilis* | 9,723 | 8,344 | 1,379 | 7,003 | 116 | 1.19 |
| *A. protothecoides* | 5,710 | 5,245 | 465 | 4,916 | 18 | 1.07 |
| *C. reinhardtii* | 19,528 | 14,984 | 4,544 | 8,012 | 1,348 | 1.87 |
| *M. commoda* | 9,954 | 7,713 | 2,241 | 6,882 | 108 | 1.12 |
| *O. tauri* | 7,709 | 6,522 | 1,187 | 6,017 | 64 | 1.08 |
| *P. coloniale* | 7,133 | 5,891 | 1,242 | 5,351 | 50 | 1.1 |
| *P.wickerhamii* strain ATCC 16529 | 6,079 | 5,798 | 281 | 5,361 | 3 | 1.08 |
| *P.wickerhamii* strain S1 | 5,694 | 5,441 | 253 | 5,151 | 8 | 1.06 |
| *P.wickerhamii* strain S931 | 5,704 | 5,493 | 211 | 5,113 | 5 | 1.07 |

# Table S12 KEGG pathway enrichment annotation in expansion of *P.wickerhamii* species

Table S12. KEGG pathway enrichment annotation in expansion of *P.wickerhamii* species

| **Genes** | **Pathway_level1** | **Pathway_level2** | **Pathway** | **PathwayID** |
| --- | --- | --- | --- | --- |
| Maker00001096 | Environmental Information Processing | Membrane transport | ABC transporters | ko02010 |
| Maker00000018 | Metabolism | Amino acid metabolism | Alanine, aspartate and glutamate metabolism | ko00250 |
| Maker00001552 | Metabolism | Carbohydrate metabolism | Amino sugar and nucleotide sugar metabolism | ko00520 |
| Maker00001068 | Genetic Information Processing | Translation | Aminoacyl-tRNA biosynthesis | ko00970 |
| Maker00004812 | Genetic Information Processing | Translation | Aminoacyl-tRNA biosynthesis | ko00970 |
| Maker00002802 | Metabolism | Amino acid metabolism | Arginine biosynthesis | ko00220 |
| Maker00001552 | Metabolism | Carbohydrate metabolism | Ascorbate and aldarate metabolism | ko00053 |
| Maker00003246 | Genetic Information Processing | Transcription | Basal transcription factors | ko03022 |
| Maker00003958 | Genetic Information Processing | Replication and repair | Base excision repair | ko03410 |
| Maker00000383 | Metabolism | Global and overview maps | Biosynthesis of amino acids | ko01230 |
| Maker00002537 | Metabolism | Global and overview maps | Biosynthesis of amino acids | ko01230 |
| Maker00002802 | Metabolism | Global and overview maps | Biosynthesis of amino acids | ko01230 |
| Maker00005452 | Metabolism | Global and overview maps | Biosynthesis of amino acids | ko01230 |
| Maker00000244 | Metabolism | Global and overview maps | Biosynthesis of secondary metabolites | ko01110 |
| Maker00000383 | Metabolism | Global and overview maps | Biosynthesis of secondary metabolites | ko01110 |
| Maker00001354 | Metabolism | Global and overview maps | Biosynthesis of secondary metabolites | ko01110 |
| Maker00002180 | Metabolism | Global and overview maps | Biosynthesis of secondary metabolites | ko01110 |
| Maker00002537 | Metabolism | Global and overview maps | Biosynthesis of secondary metabolites | ko01110 |
| Maker00002779 | Metabolism | Global and overview maps | Biosynthesis of secondary metabolites | ko01110 |
| Maker00002802 | Metabolism | Global and overview maps | Biosynthesis of secondary metabolites | ko01110 |
| Maker00003996 | Metabolism | Global and overview maps | Biosynthesis of secondary metabolites | ko01110 |
| Maker00004816 | Metabolism | Global and overview maps | Biosynthesis of secondary metabolites | ko01110 |
| Maker00005396 | Metabolism | Global and overview maps | Biosynthesis of secondary metabolites | ko01110 |
| Maker00005452 | Metabolism | Global and overview maps | Biosynthesis of secondary metabolites | ko01110 |
| Maker00005396 | Metabolism | Energy metabolism | Carbon fixation in photosynthetic organisms | ko00710 |
| Maker00002180 | Metabolism | Global and overview maps | Carbon metabolism | ko01200 |
| Maker00002779 | Metabolism | Global and overview maps | Carbon metabolism | ko01200 |
| Maker00005396 | Metabolism | Global and overview maps | Carbon metabolism | ko01200 |
| Maker00005396 | Metabolism | Carbohydrate metabolism | Citrate cycle (TCA cycle) | ko00020 |
| Maker00000244 | Metabolism | Amino acid metabolism | Cysteine and methionine metabolism | ko00270 |
| Maker00002537 | Metabolism | Amino acid metabolism | Cysteine and methionine metabolism | ko00270 |
| Maker00005396 | Metabolism | Amino acid metabolism | Cysteine and methionine metabolism | ko00270 |
| Maker00005452 | Metabolism | Amino acid metabolism | Cysteine and methionine metabolism | ko00270 |
| Maker00004462 | Genetic Information Processing | Replication and repair | DNA replication | ko03030 |
| Maker00003454 | Cellular Processes | Transport and catabolism | Endocytosis | ko04144 |
| Maker00004027 | Cellular Processes | Transport and catabolism | Endocytosis | ko04144 |
| Maker00003689 | Metabolism | Metabolism of cofactors and vitamins | Folate biosynthesis | ko00790 |
| Maker00004921 | Metabolism | Metabolism of cofactors and vitamins | Folate biosynthesis | ko00790 |
| Maker00004298 | Metabolism | Carbohydrate metabolism | Fructose and mannose metabolism | ko00051 |
| Maker00002552 | Metabolism | Metabolism of other amino acids | Glutathione metabolism | ko00480 |
| Maker00004312 | Metabolism | Metabolism of other amino acids | Glutathione metabolism | ko00480 |
| Maker00002779 | Metabolism | Amino acid metabolism | Glycine, serine and threonine metabolism | ko00260 |
| Maker00003343 | Metabolism | Amino acid metabolism | Glycine, serine and threonine metabolism | ko00260 |
| Maker00005452 | Metabolism | Amino acid metabolism | Glycine, serine and threonine metabolism | ko00260 |
| Maker00002180 | Metabolism | Carbohydrate metabolism | Glycolysis / Gluconeogenesis | ko00010 |
| Maker00002180 | Metabolism | Carbohydrate metabolism | Glyoxylate and dicarboxylate metabolism | ko00630 |
| Maker00002779 | Metabolism | Carbohydrate metabolism | Glyoxylate and dicarboxylate metabolism | ko00630 |
| Maker00005396 | Metabolism | Carbohydrate metabolism | Glyoxylate and dicarboxylate metabolism | ko00630 |
| Maker00002537 | Metabolism | Biosynthesis of other secondary metabolites | Isoquinoline alkaloid biosynthesis | ko00950 |
| Maker00005452 | Metabolism | Amino acid metabolism | Lysine biosynthesis | ko00300 |
| Maker00003565 | Environmental Information Processing | Signal transduction | MAPK signaling pathway - plant | ko04016 |
| Maker00000244 | Metabolism | Global and overview maps | Metabolic pathways | ko01100 |
| Maker00000383 | Metabolism | Global and overview maps | Metabolic pathways | ko01100 |
| Maker00000604 | Metabolism | Global and overview maps | Metabolic pathways | ko01100 |
| Maker00001332 | Metabolism | Global and overview maps | Metabolic pathways | ko01100 |
| Maker00001552 | Metabolism | Global and overview maps | Metabolic pathways | ko01100 |
| Maker00002091 | Metabolism | Global and overview maps | Metabolic pathways | ko01100 |
| Maker00002180 | Metabolism | Global and overview maps | Metabolic pathways | ko01100 |
| Maker00002537 | Metabolism | Global and overview maps | Metabolic pathways | ko01100 |
| Maker00002779 | Metabolism | Global and overview maps | Metabolic pathways | ko01100 |
| Maker00002802 | Metabolism | Global and overview maps | Metabolic pathways | ko01100 |
| Maker00003343 | Metabolism | Global and overview maps | Metabolic pathways | ko01100 |
| Maker00003689 | Metabolism | Global and overview maps | Metabolic pathways | ko01100 |
| Maker00003996 | Metabolism | Global and overview maps | Metabolic pathways | ko01100 |
| Maker00004171 | Metabolism | Global and overview maps | Metabolic pathways | ko01100 |
| Maker00004426 | Metabolism | Global and overview maps | Metabolic pathways | ko01100 |
| Maker00004816 | Metabolism | Global and overview maps | Metabolic pathways | ko01100 |
| Maker00004921 | Metabolism | Global and overview maps | Metabolic pathways | ko01100 |
| Maker00005396 | Metabolism | Global and overview maps | Metabolic pathways | ko01100 |
| Maker00005452 | Metabolism | Global and overview maps | Metabolic pathways | ko01100 |
| Maker00004462 | Genetic Information Processing | Replication and repair | Mismatch repair | ko03430 |
| Maker00005452 | Metabolism | Biosynthesis of other secondary metabolites | Monobactam biosynthesis | ko00261 |
| Maker00000476 | Genetic Information Processing | Translation | mRNA surveillance pathway | ko03015 |
| Maker00000769 | Genetic Information Processing | Translation | mRNA surveillance pathway | ko03015 |
| Maker00000960 | Genetic Information Processing | Translation | mRNA surveillance pathway | ko03015 |
| Maker00004645 | Genetic Information Processing | Translation | mRNA surveillance pathway | ko03015 |
| Maker00004662 | Genetic Information Processing | Translation | mRNA surveillance pathway | ko03015 |
| Maker00004816 | Genetic Information Processing | Translation | mRNA surveillance pathway | ko03015 |
| Maker00003246 | Genetic Information Processing | Replication and repair | Nucleotide excision repair | ko03420 |
| Maker00004418 | Genetic Information Processing | Replication and repair | Nucleotide excision repair | ko03420 |
| Maker00004462 | Genetic Information Processing | Replication and repair | Nucleotide excision repair | ko03420 |
| Maker00003689 | Metabolism | Metabolism of cofactors and vitamins | One carbon pool by folate | ko00670 |
| Maker00002091 | Metabolism | Energy metabolism | Oxidative phosphorylation | ko00190 |
| Maker00004171 | Metabolism | Energy metabolism | Oxidative phosphorylation | ko00190 |
| Maker00001552 | Metabolism | Carbohydrate metabolism | Pentose and glucuronate interconversions | ko00040 |
| Maker00001450 | Cellular Processes | Transport and catabolism | Peroxisome | ko04146 |
| Maker00001815 | Cellular Processes | Transport and catabolism | Phagosome | ko04145 |
| Maker00004171 | Cellular Processes | Transport and catabolism | Phagosome | ko04145 |
| Maker00002537 | Metabolism | Amino acid metabolism | Phenylalanine metabolism | ko00360 |
| Maker00000383 | Metabolism | Amino acid metabolism | Phenylalanine, tyrosine and tryptophan biosynthesis | ko00400 |
| Maker00002537 | Metabolism | Amino acid metabolism | Phenylalanine, tyrosine and tryptophan biosynthesis | ko00400 |
| Maker00004816 | Metabolism | Biosynthesis of other secondary metabolites | Phenylpropanoid biosynthesis | ko00940 |
| Maker00004738 | Genetic Information Processing | Folding, sorting and degradation | Protein export | ko03060 |
| Maker00001815 | Genetic Information Processing | Folding, sorting and degradation | Protein processing in endoplasmic reticulum | ko04141 |
| Maker00002858 | Genetic Information Processing | Folding, sorting and degradation | Protein processing in endoplasmic reticulum | ko04141 |
| Maker00004738 | Genetic Information Processing | Folding, sorting and degradation | Protein processing in endoplasmic reticulum | ko04141 |
| Maker00000604 | Metabolism | Nucleotide metabolism | Purine metabolism | ko00230 |
| Maker00000635 | Metabolism | Nucleotide metabolism | Pyrimidine metabolism | ko00240 |
| Maker00001332 | Metabolism | Nucleotide metabolism | Pyrimidine metabolism | ko00240 |
| Maker00003689 | Metabolism | Nucleotide metabolism | Pyrimidine metabolism | ko00240 |
| Maker00000029 | Metabolism | Carbohydrate metabolism | Pyruvate metabolism | ko00620 |
| Maker00002180 | Metabolism | Carbohydrate metabolism | Pyruvate metabolism | ko00620 |
| Maker00005396 | Metabolism | Carbohydrate metabolism | Pyruvate metabolism | ko00620 |
| Maker00002693 | Genetic Information Processing | Translation | Ribosome | ko03010 |
| Maker00003577 | Genetic Information Processing | Translation | Ribosome | ko03010 |
| Maker00003927 | Genetic Information Processing | Translation | Ribosome | ko03010 |
| Maker00003955 | Genetic Information Processing | Translation | Ribosome | ko03010 |
| Maker00004552 | Genetic Information Processing | Translation | Ribosome | ko03010 |
| Maker00004733 | Genetic Information Processing | Translation | Ribosome | ko03010 |
| Maker00000265 | Genetic Information Processing | Translation | Ribosome biogenesis in eukaryotes | ko03008 |
| Maker00003482 | Genetic Information Processing | Translation | Ribosome biogenesis in eukaryotes | ko03008 |
| Maker00005333 | Genetic Information Processing | Translation | Ribosome biogenesis in eukaryotes | ko03008 |
| Maker00000767 | Genetic Information Processing | Folding, sorting and degradation | RNA degradation | ko03018 |
| Maker00000769 | Genetic Information Processing | Translation | RNA transport | ko03013 |
| Maker00000960 | Genetic Information Processing | Translation | RNA transport | ko03013 |
| Maker00001065 | Genetic Information Processing | Translation | RNA transport | ko03013 |
| Maker00002769 | Genetic Information Processing | Translation | RNA transport | ko03013 |
| Maker00002924 | Genetic Information Processing | Translation | RNA transport | ko03013 |
| Maker00004645 | Genetic Information Processing | Translation | RNA transport | ko03013 |
| Maker00004662 | Genetic Information Processing | Translation | RNA transport | ko03013 |
| Maker00004816 | Genetic Information Processing | Translation | RNA transport | ko03013 |
| Maker00000540 | Genetic Information Processing | Transcription | Spliceosome | ko03040 |
| Maker00004914 | Genetic Information Processing | Transcription | Spliceosome | ko03040 |
| Maker00003996 | Metabolism | Carbohydrate metabolism | Starch and sucrose metabolism | ko00500 |
| Maker00001354 | Metabolism | Lipid metabolism | Steroid biosynthesis | ko00100 |
| Maker00002537 | Metabolism | Biosynthesis of other secondary metabolites | Tropane, piperidine and pyridine alkaloid biosynthesis | ko00960 |
| Maker00002537 | Metabolism | Amino acid metabolism | Tryptophan metabolism | ko00380 |
| Maker00002537 | Metabolism | Amino acid metabolism | Tyrosine metabolism | ko00350 |
| Maker00004426 | Metabolism | Amino acid metabolism | Tyrosine metabolism | ko00350 |
| Maker00002537 | Metabolism | Metabolism of cofactors and vitamins | Ubiquinone and other terpenoid-quinone biosynthesis | ko00130 |
| Maker00000634 | Genetic Information Processing | Folding, sorting and degradation | Ubiquitin mediated proteolysis | ko04120 |
| Maker00003970 | Genetic Information Processing | Folding, sorting and degradation | Ubiquitin mediated proteolysis | ko04120 |
| Maker00004418 | Genetic Information Processing | Folding, sorting and degradation | Ubiquitin mediated proteolysis | ko04120 |

# Table S13 The SNP information in *P.wickerhamii* genomes.

Table S13. The SNP information in *P. wickerhamii* genomes.

| **SNP type** | **S1 vs S931** | | **S1 vs ATCC 16529** | | **ATCC 16529 vs S931** | |
| --- | --- | --- | --- | --- | --- | --- |
|  | **Number** | **Percent** | **Number** | **Percent** | **Number** | **Percent** |
| **A<->G** | 28,022 | 29.74% | 66,385 | 30.82% | 98,667 | 29.25% |
| **C<->T** | 28,394 | 30.13% | 66,959 | 31.09% | 98,977 | 29.35% |
| **Transitions** | 56,416 | 59.87% | 133,344 | 61.91% | 197,644 | 58.60% |
| **A<->C** | 9,453 | 10.03% | 20,441 | 9.49% | 34,677 | 10.28% |
| **A<->T** | 6,611 | 7.02% | 12,506 | 5.81% | 24,561 | 7.28% |
| **C<->G** | 12,527 | 13.29% | 29,031 | 13.48% | 46,190 | 13.69% |
| **G<->T** | 9,229 | 9.79% | 20,066 | 9.32% | 34,210 | 10.14% |
| **Transversions** | 37,820 | 40.13% | 82,044 | 38.09% | 139,638 | 41.40% |
| **Transition/Transversion** | 1.492 |  | 1.625 |  | 1.415 |  |
| **Total** | **94,236** |  | **215,388** |  | **337,282** |  |
